# Optimized RNA-targeting CRISPR/Cas13d technology outperforms shRNA in identifying functional circRNAs

**DOI:** 10.1186/s13059-021-02263-9

**Published:** 2021-01-21

**Authors:** Yang Zhang, Tuan M. Nguyen, Xiao-Ou Zhang, Limei Wang, Tin Phan, John G. Clohessy, Pier Paolo Pandolfi

**Affiliations:** 1 Cancer Research Institute, Beth Israel Deaconess Cancer Center, Department of Medicine and Pathology, Beth Israel Deaconess Medical Center, Harvard Medical School, Boston, MA 02215 USA; 2 Ludwig Center at Harvard, Harvard Medical School, Boston, MA 02215 USA; 3 Present address: Section on Integrative Physiology and Metabolism, Joslin Diabetes Center, Harvard Medical School, Boston, MA 02215 USA; 4 Present address: Chemical Biology and Therapeutics Science, Broad Institute of MIT and Harvard, Cambridge, MA 02142 USA; 5 Program in Bioinformatics and Integrative Biology, University of Massachusetts Medical School, Worcester, MA 01605 USA; 6 Preclinical Murine Pharmacogenetics Facility and Mouse Hospital, Beth Israel Deaconess Medical Center, Harvard Medical School, Boston, MA 02215 USA; 7 Department of Molecular Biotechnology and Health Sciences, University of Turin, 10126 Turin, Italy; 8 Renown Institute for Cancer, Nevada System of Higher Education, Reno, NV 89502 USA

**Keywords:** circRNAs, CRISPR/Cas13d system, High-throughput screening

## Abstract

Short hairpin RNAs (shRNAs) are used to deplete circRNAs by targeting back-splicing junction (BSJ) sites. However, frequent discrepancies exist between shRNA-mediated circRNA knockdown and the corresponding biological effect, querying their robustness. By leveraging CRISPR/Cas13d tool and optimizing the strategy for designing single-guide RNAs against circRNA BSJ sites, we markedly enhance specificity of circRNA silencing. This specificity is validated in parallel screenings by shRNA and CRISPR/Cas13d libraries. Using a CRISPR/Cas13d screening library targeting > 2500 human hepatocellular carcinoma-related circRNAs, we subsequently identify a subset of sorafenib-resistant circRNAs. Thus, CRISPR/Cas13d represents an effective approach for high-throughput study of functional circRNAs.

**Supplementary Information:**

The online version contains supplementary material available at 10.1186/s13059-021-02263-9.

## Background

Circular RNAs (circRNAs) are covalently closed, single-stranded transcripts, which are produced by back-splicing of precursor mRNAs (pre-mRNAs). Thousands of circRNAs have been discovered across species with cell-type- and tissue-specific expression patterns [1–4]. However, the functional repertoire of circRNAs remains mostly uncharacterized to date, which is mainly due to the unique properties of circRNAs and limitations of current approaches in circRNA studies [5, 6]. The rapid development of CRISPR-Cas9-based genomics screens has dramatically enhanced the speed and precision of functional characterization of both coding genes and linear non-coning RNAs [7]. Nevertheless, the majority of circRNAs are generated from protein-coding genes [8], and hence, the sequences of circRNAs are completely overlapping with their cognate linear RNAs processed from the same pre-mRNAs. Such features of circRNAs largely limit the application of Cas9 and its variant-mediated gene manipulations in understanding the functional relevance of circRNAs. Knockout of circRNAs is another loss of function (LOF) assay to study the function of circRNAs. The assay can be achieved by depleting the complementary sequences (CSs) in flanking introns [9] since the biogenesis of circRNAs is enhanced by RNA pairing of intronic CSs [10, 11]. However, the complexity of complementary sequence-mediated exon circularization [11, 12] makes it difficult to apply this approach to annotate the functions of circRNAs at a genome-wide scale. Therefore, although it is known that RNA interference (RNAi) has widespread non-specific transcript silencing [13, 14], RNAi-mediated degradation is still the major modality to date to silence circRNAs by targeting the unique BSJ site of circRNAs. Unfortunately, the requirement of designing shRNA/siRNA targeting BSJ sites limits the possibility to utilize multiple shRNAs/siRNAs with distinct coverage to rule out the potential off-target effects [5]. Recently, shRNA-based functional screen has been employed to understand circRNA essentiality [15]. However, in our study, we observed frequent discrepancies between shRNA-mediated circRNA knockdown efficiency and the corresponding biological effect on cell proliferation (see below), raising concerns about the robustness of using RNAi to study the function of circRNAs. Thus, the development of additional methods to achieve specific and efficient knockdown of circRNAs remains an important priority. In this study, we optimized the strategy of designing CRISPR/Cas13d gRNAs to specifically and effectively silence circRNAs, which are more complex to selectively target than linear transcripts. We also leveraged the optimized system for high-throughput circRNA functional screening and compared its precision with shRNA-based screening. Using a Cas13d library targeting a large number of human hepatocellular carcinoma (HCC)- related circRNAs, we successfully identified functional circRNAs, whose inhibition increased the therapeutic efficacy of the multikinase inhibitor, sorafenib. Collectively, the optimized Cas13d platform proved to be more robust than shRNAs in identifying bona fide functional circRNAs.

## Results

### Targeting conserved HCC circRNAs with shRNAs

Given the tissue-specific expression pattern of circRNAs, in this study, we focused on human HCC-related circRNAs. We re-analyzed total RNA sequencing (rRNA-depleted RNA-seq) data of paired primary tumors and adjacent normal tissues from 20 HCC patients [16] with CIRCexplorer2 to determine circRNA expression (Additional file 1: Figure S1a,b, Additional file 2: Table S1, see details in “Methods”). We found 134 highly expressed circRNAs, among which 20 differentially expressed circRNAs were conserved between human and mouse (Additional file 1: Figure S1b,c, Additional file 3: Table S2). Top 10 conserved circRNAs (Additional file 1: Figure S1c) were selected for further characterization. RT-PCR with divergent primers across the BSJ sites followed by Sanger sequencing confirmed the junction sites (Additional file 1: Figure S1d, e, g). RNase R resistance confirmed the circular structure of 9 out of the 10 conserved circRNAs, except for circARHGAP5 (or circArhgap5 in mouse) (Additional file 1: Figure S1f, h), which might originate from an aberrant splicing event (Additional file 1: Figure S1i). Moreover, most circRNAs predominantly localized in the cytoplasm, except two circRNAs (circFBXW4 and circUBE3A) that showed half nuclear distribution (Additional file 1: Figure S1j).

To investigate the functions of these validated circRNAs, we performed cell proliferation assay upon knockdown of each circRNA with two sets of shRNAs (Additional file 1: Figure S2a). Knockdown efficiency of each shRNA was confirmed by qRT-PCR (Additional file 1: Figure S2b). Interestingly, we found that knockdown of two circRNAs, circASPH and circZNF292, led to significant decreased proliferation rate compared to control cells (Additional file 1: Figure S2c,d). However, we also noticed a dramatic difference in growth between two individual shRNAs, despite comparable knockdown efficiency (Additional file 1: Figure S2c,d). The inconsistency between shRNA knockdown efficiency and inhibition of cell proliferation compelled us to include additional experimental strategies to assess the potential essentiality of these circRNAs. Antisense locked nucleic acid (LNA) GapmerRs, which are considered to be more specific than siRNA [17, 18], were used to target circASPH. The circular structure of circASPH was further confirmed by northern blot with RNase R treatment (Additional file 1: Figure S2e). Surprisingly, despite the similar knockdown level of circASPH (Additional file 1: Figure S2f-i), LNA-mediated circASPH knockdown led to no obvious difference in the proliferation rate compared to control cells (Additional file 1: Figure S2j), raising concern about the reliability of shRNA to assess circRNA essentiality.

### Optimization of CRISPR/Cas13d system for circRNA knockdown

**Fig. 1:**
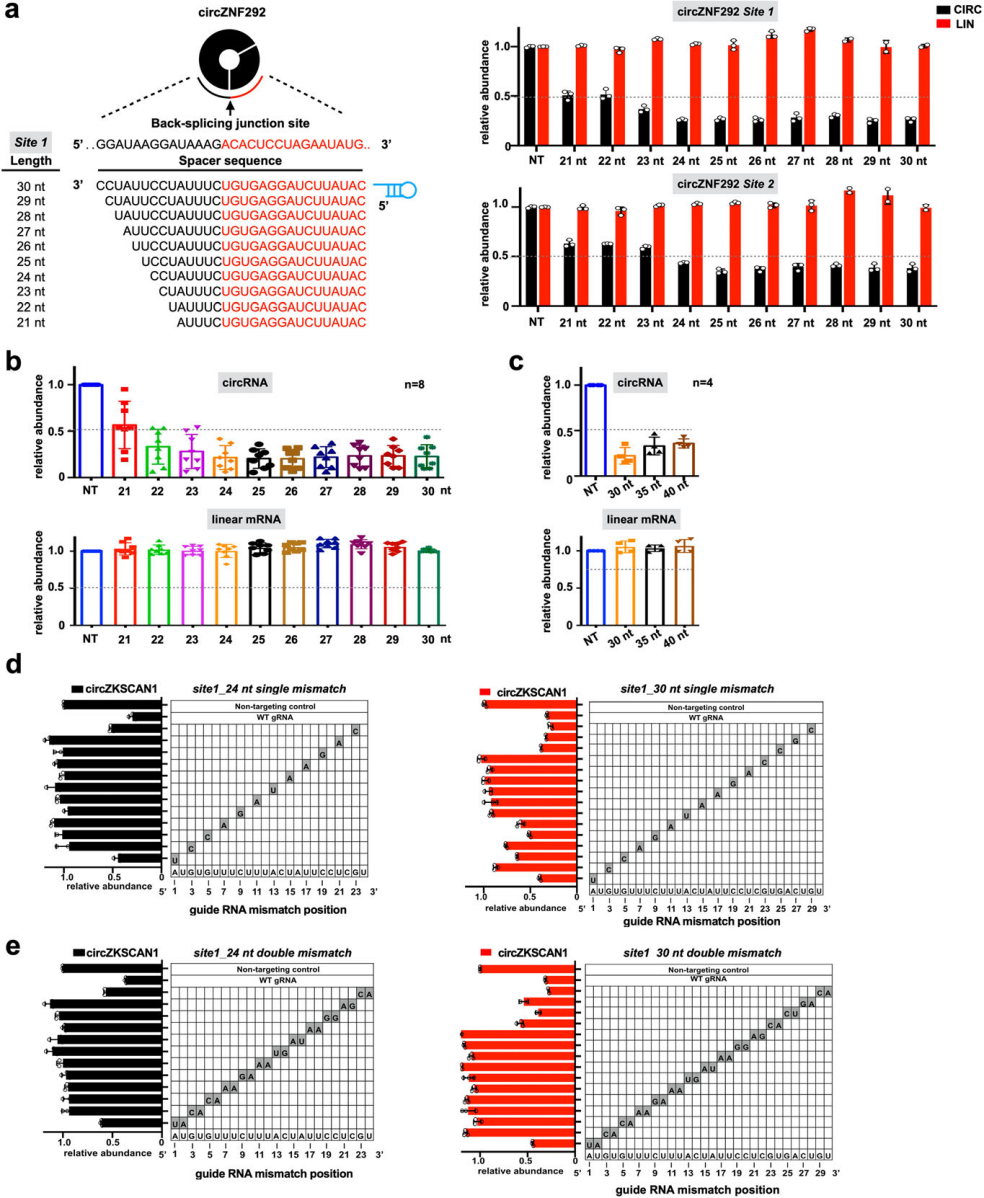
Optimization of CRISPR-Cas13d for circRNA knockdown. **a** Schematic view of the length optimization of gRNAs targeting BSJ sites of circRNAs (left panel) and the corresponding knockdown efficiency with different lengths of gRNAs (right panel). Bar plots showing the relative expression of circZNF292 (CIRC) and its parental linear transcript (LIN) upon knockdown of circZNF292 with two gRNAs containing spacers ranging from 21 to 30 nt in length. NT, non-targeting. **b** Bar graphs showing the cumulative knockdown efficiency of different lengths of gRNAs across multiple circRNAs. Top, relative expression of targeting circRNAs. Bottom, relative expression of cognate linear mRNAs (*n* = 8 circRNA target sites, the data for each circRNA target site can be found in **a** and Additional file 1: Figure S3b). NT, non-targeting. **c** Bar graphs showing cumulative knockdown efficiency of longer gRNAs with 30 nt, 35 nt, and 40 nt spacers. Top, relative expression of targeting circRNAs. Bottom, relative expression of cognate linear mRNAs (*n* = 4 circRNA target sites, the data for each circRNA target site can be found in Additional file 1: Figure S3c). NT, non-targeting. **d** Knockdown of circZKSCAN1 evaluated with gRNAs containing 24 nt length spacer (left) or 30 nt length spacer (right) with single mismatch at varying positions across the spacer sequence. The gray boxes in the grids show the position of Watson-Crick transversion mismatches. The wild-type sequence is shown at the bottom of each grid. **e** Knockdown of circZKSCAN1 evaluated with gRNAs containing 24 nt length spacer (left) or 30 nt length spacer (right) with consecutive double mismatch at varying positions across the spacer sequence. The gray boxes in the grids show the position of Watson-Crick transversion mismatches. The wild-type sequence is shown at the bottom of each grid. The data shown are from one of two biological replicates with similar results, and error bars indicating the mean ± s.d. of three technical replicates

To address the shRNA issue and develop a more reliable knockdown tool to study the function of circRNAs, we sought to leverage the CRISPR/Cas13d system for depleting circRNAs. CRISPR/Cas13d system is a recently developed RNA-guided, RNA-targeting CRISPR system, which has been used to mediate efficient and specific knockdown of diverse linear transcripts [19]. The most effective Cas13d enzyme, CasRx paired with two distinct guide RNA architectures [19], unprocessed pre-gRNA, and mature gRNA were employed to target circRNAs (Additional file 1: Figure S3a). Compared to mature gRNAs with fixed 22 nt spacers, the transcribed pre-gRNA is processed into ~ 52 nt mature gRNAs, with a 30 nt 5′ direct repeat followed by a variable 3′ spacer ranging from 14 to 26 nt in length [19]. We found that pre-gRNA architectures, which were further processed into mature gRNAs with varying spacer lengths, mediated a more potent knockdown than gRNAs with fixed 22 nt spacers (Additional file 1: Figure S3a). Therefore, we speculated that different spacer lengths may confer different levels of circRNA knockdown. To test our prediction, we generated a series of constructs that expressed progressively shorter mature gRNAs with spacers ranging from 30 to 21 nt in length (Fig. 1a). We found that gRNAs with 24 to 30 nt of target complementarity showed comparable knockdown efficacy, whereas CasRx cleavage activity decreased when paired with gRNAs containing spacer sequences that were shorter than 23 nt (Fig. 1a,b, Additional file 1: Figure S3b). We also observed that gRNAs with more than 30 nt of target complementarity showed less efficient knockdown of circRNAs (Fig. 1c, Additional file 1: Figure S3c). To further finalize the optimal spacer length and decide if gRNAs with the longer or the shorter spacers are more specific, we evaluated the specificity of gRNAs with either 24 nt spacer (hereafter referred to as 24 nt gRNA) or 30 nt spacer (hereafter referred to as 30 nt gRNA) by assessing their sensitivity to Watson-Crick mismatches at the gRNA-circRNA interface. We generated a series of variants of 24 nt gRNAs and 30 nt gRNAs targeting circZKSCAN1 (Fig. 1d,e). These variants contained single mismatches or consecutive double mismatches at indicated positions. We found that efficient knockdown of circRNAs was, in general, less tolerant to mismatches inserted in the middle region (Fig. 1d,e). Intriguingly, 24 nt gRNA-mediated knockdown was more sensitive to both single and double mismatches compared to 30 nt gRNAs (Fig. 1d,e). These findings were also observed in another set of gRNAs targeting circZNF292 (Additional file 1: Figure S3d,e), confirming that gRNAs with 24 nt spacer length are more specific than gRNAs with 30 nt spacer length. In addition to optimizing the length of the gRNA spacer for Cas13d, we explored the effect of Cas13d-mediated circRNA knockdown using gRNAs targeting different positions surrounding the BSJ site. Five gRNAs with fixed 24 nt spacers across the BSJ sites in incremental steps were designed to target each of six different circRNAs (Additional file 1: Figure S3f). However, based on the current data (Additional file 1: Figure S3f), it is difficult to generalize the optimal position for designing gRNAs for circRNAs, likely due to the complex structure of circRNAs. Therefore, multiple gRNAs covering BSJ sites are suggested to be tested in order to ensure efficient knockdown of target circRNAs. Taken together, we successfully optimized the strategy to generate CRISPR/Cas13d gRNAs with optimal efficacy and specificity for circRNA knockdown, and gRNAs with the 24 nt spacer design were used for subsequent experiments.

To compare the specificity of the CRISPR/Cas13d system with shRNA and LNA for knocking down circRNAs, we applied the optimized Cas13d system to silencing circASPH, which showed inconsistent growth phenotypes with different shRNAs in comparison with the LNA knockdown method (Additional file 1: Figure S2c, f-j). Huh7 cells with stably expressed CasRx were transduced with gRNAs containing 24 nt spacer sequences targeting the BSJ site of circASPH. Northern blots confirmed Cas13d-mediated circASPH knockdown (Additional file 1: Figure S2k, l). Similar to LNA-mediated circASPH knockdown, no obvious difference in the proliferation rate was observed between Cas13d-mediated circASPH-silencing cells and control cells (Additional file 1: Figure S2m), demonstrating the off-target effects of shRNA for circRNA knockdown, and further proving the reliability of Cas13d in assessing the function of circRNAs.

### Optimized Cas13d mediates efficient and specific knockdown of circRNAs

**Fig. 2:**
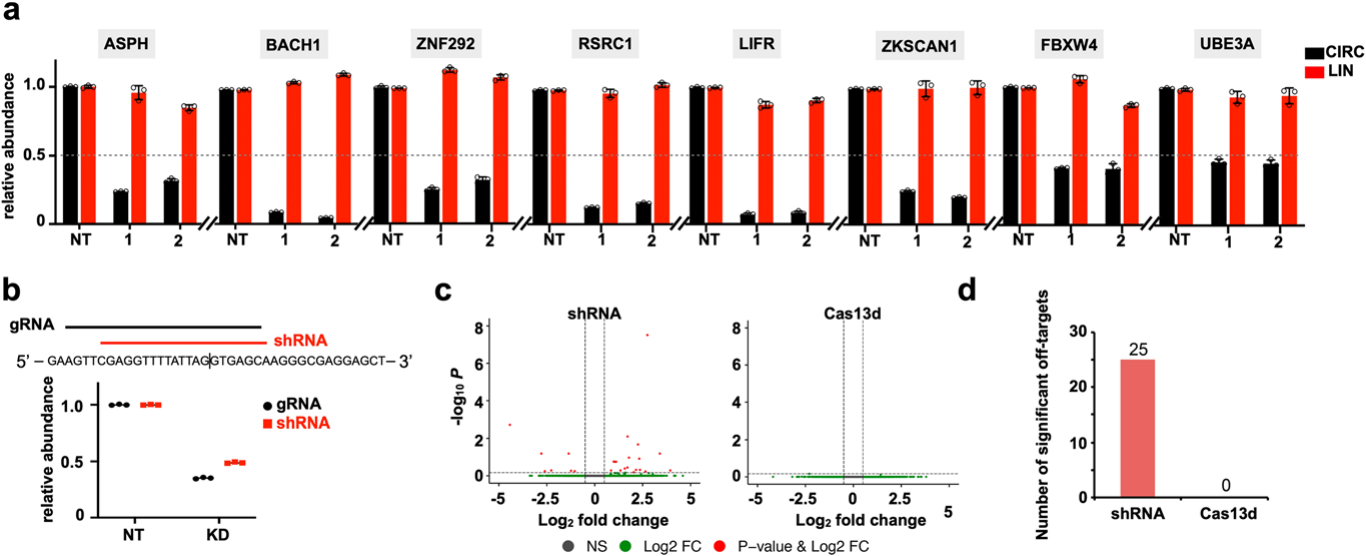
Characteristics of Cas13d-mediated circRNA knockdown. **a** Optimized Cas13d system targeting 8 circRNAs, each with 2 gRNAs. qRT-PCR for circular and linear transcripts after knockdown of circRNAs in Huh7 cells. **b** Top, schematic drawing of circEGFP-targeting guide RNA sequence and spacer position-matched shRNA. Bottom, relative circEGFP knockdown by individual position-matched gRNA and shRNA. NT, non-targeting. **c** Volcano plots of differential transcript levels between circEGFP-targeting and non-targeting shRNAs (left) or circEGFP-targeting CasRx and non-targeting guide (right) as determined by RNA sequencing. **d** Summary of significant off-target transcript perturbations by matched Cas13d gRNA and shRNA. The data shown are from one of two biological replicates with similar results, and error bars indicating the mean ± s.d. of three technical replicates

To evaluate the range of efficiency of optimized Cas13d knockdown, we designed gRNAs to target the same endogenous circRNAs that have been successfully silenced by shRNAs (Additional file 1: Figure S2b). As revealed by qRT-PCR, gRNAs showed comparable knockdown efficiency to shRNA-mediated circRNA degradation (Fig. 2a).

Importantly, we also sought to optimize the targeting of circRNAs with nuclear distribution (circFBXW4 and circUBE3A). To this end, we employed a nuclear-localized version of CasRx (CasRx with nuclear localization signal, CasRx-NLS) [19] and side-by-side compared the ability of CasRx with or without NLS to target cricRNAs with different cellular localization (Additional file 1: Figure S3g). Indeed, we found that CasRx with NLS was more efficient in targeting circRNAs with nuclear distribution (circFBXW4 and circUBE3A) (Additional file 1: Figure S3g). In contrast, for cytosolic circRNAs (circLIFR and circBACH1), CasRx without NLS mediated more robust knockdown efficiency (Additional file 1: Figure S3g). These data suggest that Cas13d is a more versatile tool than shRNA and allows for compartmentalized targeting of circRNAs.

To further assess the specificity of Cas13d *vis a vis* that of shRNA for circRNA knockdown, position-matched gRNA and shRNA were used to target circEGFP, which originated from back-splicing of an EGFP exon that was mediated by CS pairing across the flanking intron (Additional file 1: Figure S3h, Additional file 4: Table S3, see details in “Methods”). Both methods achieved comparable levels of circEGFP knockdown (Fig. 2b). Since circEGFP is not endogenous to the cell, cells with circEGFP knockdown should have similar transcriptomic profiles to cells transduced with non-targeting shRNA or gRNA. We observed that compared to Cas13d, shRNAs showed higher variability between targeting and non-targeting conditions (Fig. 2c). Differential expression analysis indicated 25 significant off-targets in shRNA condition but none in Cas13d condition (Fig. 2d). Collectively, compared to shRNA, our optimized Cas13d strategy showed comparable circRNA knockdown efficiency, but with higher specificity.

### Systematic comparison of Cas13d and shRNA functional screenings for circRNAs

**Fig. 3:**
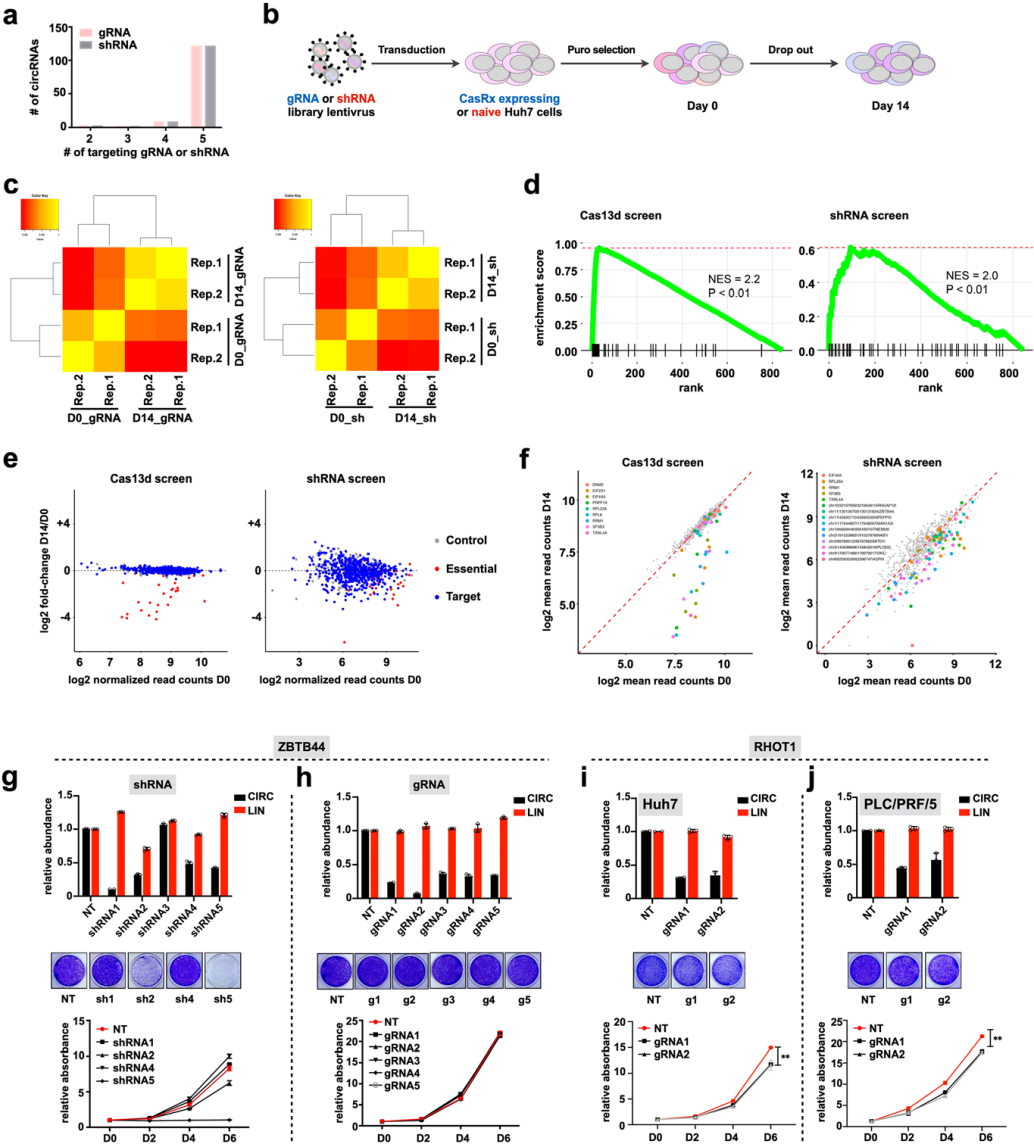
Systematic comparison of CRISPR-Cas13d and shRNA functional screenings for circRNAs. **a** Number of gRNAs and shRNAs per circRNA in the library. **b** Schematic view of the screenings. Cas13d and shRNA lentivirus libraries were infected into CasRx stably expressed Huh7 cells or naive Huh7 cells separately and selected by puromycin treatment (time zero). Puromycin-resistant cells were further cultured for 14 days. Genomic DNA was extracted at indicated time points and library representation was determined by deep sequencing. **c** Correlation heatmap showing the Pearson correlation coefficient between the levels of gRNAs/shRNAs in biological replicates of time zero samples (D0) and 14-day enrichment samples (D14) for Cas13d screen (left) and shRNA screen (right). **d** Gene set enrichment analysis (GSEA) revealed essential genes are enriched in negative selections for Cas13d screen (left) and shRNA screen (right). Essential genes serve as positive controls. The degree of enrichment is measured as normalized enrichment score (NES). **e** Scatterplots showing log_2_-transformed fold-change of gRNA/shRNA normalized read counts in D14 vs. D0 for Cas13d screen (left) and shRNA screen (right). Control, non-targeting controls; Essential, positive controls targeting known essential genes; Target, circRNAs highly expressed in HCC. **f** Scatterplots showing negatively selected gRNA/shRNAs and corresponding genes from Cas13d screen (left) and shRNA screen (right) with FDR < 0.25. CircRNAs are indicated with genomic locations and the host gene name at the end (e.g., chr10|32197099|32199491|ARHGAP12|). Positive controls only have gene names without genomic location (e.g., EIF4A3). **g**, **h** Top, relative expression levels of circZBTB44 and its parental mRNA upon knockdown of circZBTB44 by shRNAs (**g**) and gRNAs (**h**) in human Huh7 cells. Middle and bottom, proliferation rates of control and shRNA-mediated (**g**) or Cas13d-mediated (**h**) circZBTB44-silenced Huh7 cells. The number of cells was detected upon staining with crystal violet, and representative pictures are shown in the middle, while the proliferation curves are shown at the bottom. **i, j** Top, relative expression levels of circRHOT1 and its parental mRNA upon knockdown of circRHOT1 by gRNAs in Huh7 cells (**i**) or PLC/PRF/5 cells (**j**). Middle and bottom, proliferation rates of control and circRHOT1-silenced Huh7 cells (**i**) or PLC/PRF/5 cells (**j**). The number of cells was detected upon staining with crystal violet. The data shown are from one of two biological replicates with similar results, and error bars indicating the mean ± s.d. of three technical replicates. *p < 0.05, **p < 0.01, ***p < 0.001 (unpaired Student’s *t* test). ns, not significant

We further evaluated the capacity of CRISPR/Cas13d system to screen for essential circRNAs in a high-throughput manner. We performed in parallel both Cas13d and shRNA screenings in Huh7 cells in order to systematically compare the two systems’ abilities to identify circRNAs that are essential for cell growth (see details in “Methods”). Briefly, position-matched gRNAs and shRNAs were designed to target the BSJ sites of 134 highly expressed circRNAs (Fig. 3a, Additional file 1: Figure S4a, Additional files 5, 6: Table S4, Table S5). gRNA and shRNA libraries were lentivirally infected into Huh7 cells and screened for gene essentiality over a 14-day period (Fig. 3b). PCR-amplified barcode-gRNAs or shRNAs from genomic DNA of cells before and after screening were subjected to deep sequencing. Overall, the read distribution of duplicated screens within each condition showed a high level of correlation for both gRNAs and shRNAs (Fig. 3c, Additional file 1: Figure S4b). To identify the top hits from the screens, we processed our sequencing data using MAGeCK algorithm (v0.5.8). Gene set enrichment analysis (GSEA) showed that both gRNAs and shRNAs targeting positive controls (10 known essential linear transcripts [20]) were significantly enriched in the ranked list of negatively selected gRNAs or shRNAs (Fig. 3d), suggesting that these two parallel screens performed as intended. However, compared to non-targeting control gRNAs in Cas13d library, non-targeting control shRNAs had a much higher level of variation (Fig. 3e, Additional file 1: Figure S4c). The observed high level of correlation between duplicated screens rules out the possibility that the high level of variation was due to technical variation. Therefore, this variation in non-targeting controls is more likely due to shRNA’s off-target effects. In contrast, non-targeting control gRNAs have a much narrower range of variation (Fig. 3e, Additional file 1: Figure S4c), confirming Cas13d’s high level of specificity. For circRNAs, MAGeCK identified 10 negatively selected circRNAs with statistical significance (false discovery rate (FDR) < 0.25) from shRNA-based screen (Fig. 3f, Additional file 7: Table S6), including circASPH that was tested in Additional file 1: Figure S2. Six of the remaining candidates were resistant to RNase R treatment (Additional file 1: Figure S5a), confirming their existence as circRNAs. These 6 circRNA candidates were further validated for their essentiality in conferring growth in Huh7 cells. We performed cell proliferation assays upon knockdown of each circRNA with five individual shRNAs presented in the library. Knockdown efficiency of each shRNA was confirmed by qRT-PCR, and most of them resulted in > 60% reduction of circRNA abundance (Fig. 3g, Additional file 1: Figure S5b, f, j, n, r). However, we noticed that several circRNA-targeting shRNAs decreased the counterpart linear transcripts as well, especially for circNPEPPS (Additional file 1: Figure S5f). Notably, similar to circASPH (Additional file 1: Figure S2c), the inconsistency between shRNA-mediated circRNA knockdown efficiency and effect on cellular proliferation rate was detected in 6 out of 6 tested circRNAs (Fig. 3g, Additional file 1: Figure S5b-u), suggesting widespread off-target effects of shRNA in circRNA knockdown. To confirm that the shRNA screen identified false positive essential circRNAs, we used position-matched gRNAs from the Cas13d screening library to target the same circRNAs. Consistent with our prediction, no obvious change in the cell proliferation rate was observed in Cas13d-mediated circRNA knockdown cells compared to control cells, despite the comparable levels of circRNA knockdown by Cas13d and shRNA (Fig. 3h, Additional file 1: Figure S5b-u). All of these 6 tested circRNAs were also identified as essential circRNAs through a second independent shRNA screening with higher read depth, ruling out technical variance as being the source of the high false positive rate of the shRNA-based screening (Additional file 1: Figure S6). Taken together, these data corroborate the high false positive rate of shRNA-based screening for identifying essential circRNAs. Thus, further optimization for the shRNA design to increase the on-target specificity is still required.

We analyzed Cas13d screening sequencing data using the same algorithm. Positive control gRNAs were significantly depleted as expected given the essential roles of their targets, whereas none of gRNAs targeting circRNAs were significantly depleted during the screening (Fig. 3f, Additional file 8: Table S7). One possible explanation could be the small size of the library (targeting 134 circRNAs), limiting the probability of identifying essential circRNAs. However, none of the false positive candidates identified in the shRNA screening was detected in the Cas13d screening, suggesting that our Cas13d screening platform has a much lower false positive rate compared with the conventional shRNA screening platform.

To evaluate the ability of Cas13d to identify bona fide essential circRNAs, we used our optimized Cas13d system to target previously reported essential circRNAs. CircRHOT1 knockout, by depleting flanking CSs, was shown to suppress HCC’s cell proliferation [21]. Knockdown of circRHOT1 via Cas13d in two HCC cell lines, Huh7 and PLC/PRF/5, inhibited cell proliferation (Fig. 3i, j). CircHIPK3 is another circRNA that is known to promote proliferation of the colon cancer cell line HCT116 [22]. Knockdown of circHIPK3, by Cas13d with three different gRNAs (Additional file 1: Figure S7a), indeed inhibited HCT116 cell proliferation (Additional file 1: Figure S7b, c). Moreover, since CDR1as functions as miR-7 ceRNA [2, 23], established miR-7 targets (*SNCA and IRS2*) were downregulated by miR-7 in Cas13d-mediated CDR1as-silenced cells compared to control cells (Additional file 1: Figure S7d, e). Taken together, these results demonstrate the ability of Cas13d to identify bona fide functional circRNAs.

### Larger Cas13d library screening identifies sorafenib-resistant circRNAs

**Fig. 4:**
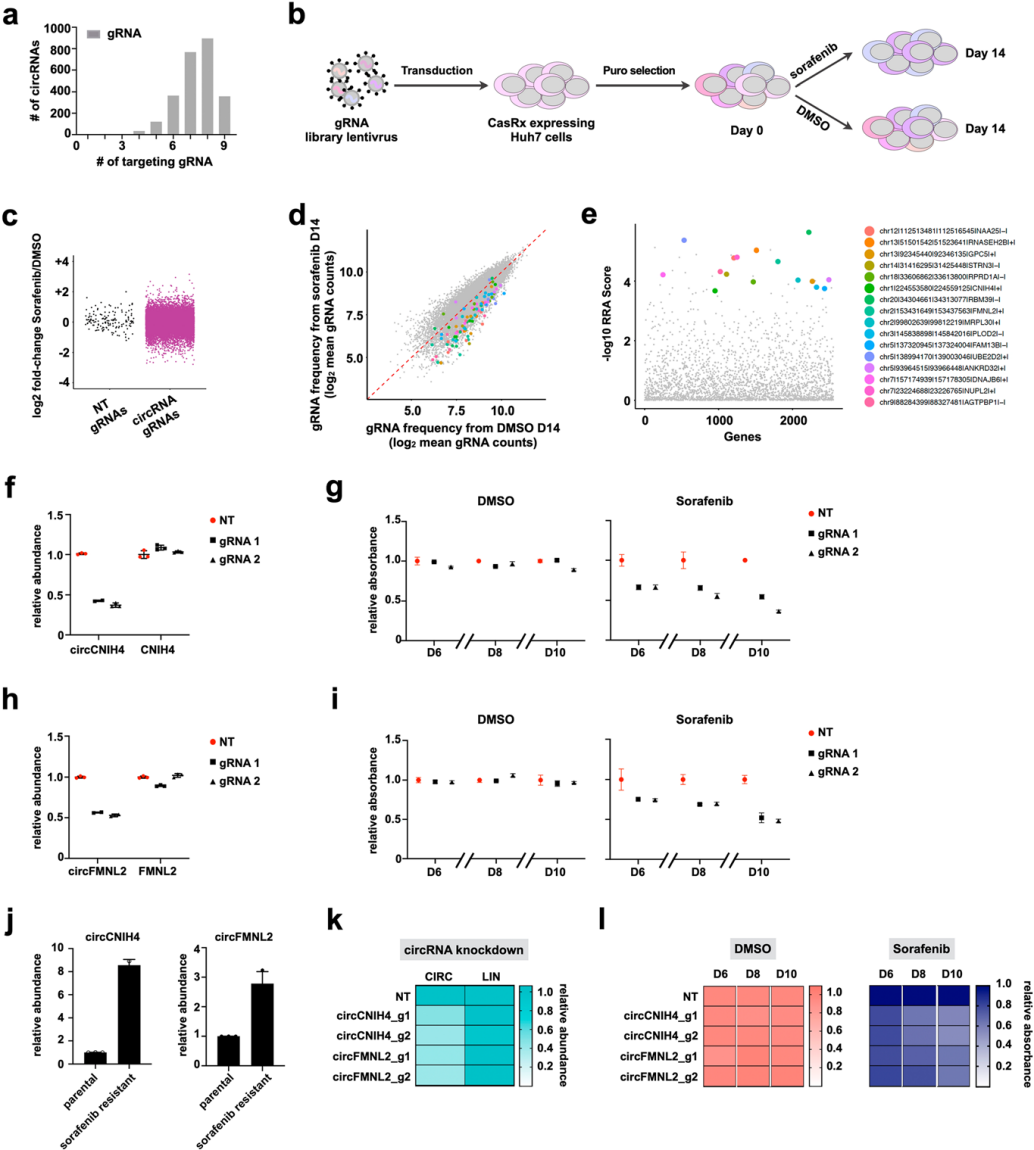
Larger Cas13d library screening identified sorafenib-resistant circRNAs. **a** Number of gRNAs per circRNA in the library. **b** Schematic view of the drug selection screening. Cas13d lentivirus libraries were infected into CasRx stably expressed Huh7 cells then selected with puromycin treatment (time zero). Puromycin-resistant cells were further treated with DMSO (vehicle) or sorafenib for 14 days. Genomic DNA was extracted at day 14, and library representation was determined by deep sequencing. **c** Plot of fold-change of NT and circRNA gRNAs between DMSO- and sorafenib-treated samples. NT, non-targeting. **d** Scatterplot showing depletion of specific gRNAs after sorafenib treatment. Color dots indicate gRNAs corresponding with experimentally validated circRNAs as shown in Additional file 1: Figure S8f, g. **e** The robust rank aggregation (RRA) scores of top negatively selected circRNAs calculated by MAGeCK. Validated sorafenib-resistant circRNAs are labeled in color dots. CircRNAs are indicated with genomic locations and the host gene symbols at the end (e.g., chr12|112513481|112516545|NAA25|-|). **f** Relative expression levels of circCNIH4 and its parental mRNA upon knockdown of circCNIH4 by two independent gRNAs. **g** Knockdown of circCNIH4 sensitized Huh7 cells to sorafenib treatment. Knockdown of circCNIH4 significantly suppressed cell proliferation in the presence of sorafenib. Cell proliferation was measured by crystal violet staining, and absorbance at 590 nm of the treated cells from each group (day 6, day 8, day 10) was normalized to NT of the same day. NT, non-targeting. **h** Relative expression levels of circFMNL2 and its parental mRNA upon knockdown of circFMNL2 by two independent gRNAs. **i** Knockdown of circFMNL2 sensitized Huh7 cells to sorafenib treatment. Knockdown of circFMNL2 significantly suppressed cell proliferation in the presence of sorafenib. Cell proliferation was measured by crystal violet staining, and absorbance at 590 nm of the treated cells from each group (day 6, day 8, day 10) was normalized to NT of the same day. NT, non-targeting. **j** Relative expression levels of circCNIH4 and circFMNL2 in parental and sorafenib-resistant Huh7 cells. **k** Heatmap display of the relative expression levels of circCNIH4, circFMNL2 and their parental mRNAs upon knockdown of circCNIH4 and circFMNL2 by gRNAs in sorafenib-resistant Huh7 cells. **l** Knockdown of circCNIH4 or circFMNL2 sensitized sorafenib-resistant Huh7 cells to sorafenib treatment. Heatmap display of the relative cell proliferation of control and Cas13d-mediated circRNA-silenced Huh7 cells treated with either DMSO or sorafenib. Cell proliferation was measured by crystal violet staining, and absorbance at 590 nm of the treated cells from each group (day 6, day 8, day10) was normalized to NT of the same day. NT, non-targeting

To generalize the application of circRNA-targeting Cas13d libraries and further evaluate the efficacy of Cas13d screening in identifying functional circRNAs, we generated a larger Cas13d library that target BSJ sites of 2543 human HCC-related circRNAs based on the RNA sequencing data from 20 HCC patients (Fig. 4a, Additional file 1: Figure S8a, Additional file 9: Table S8). For advanced HCC, the first-line therapy is sorafenib, which is an oral multikinase inhibitor [24]. However, resistance to sorafenib frequently occurs and limits the benefit of such therapeutic option. Recently, genome-wide expression pattern of circRNAs in sorafenib-resistant HCC cells has been analyzed, and over 1000 differentially expressed circRNAs have been identified [25], indicating a potential role of circRNAs in the development of sorafenib resistance. To test this hypothesis, we performed drug selection screening with the larger Cas13d library to identify circRNAs, whose inhibition could increase the therapeutic efficacy of sorafenib in Huh7 cells (Fig. 4b). After 14 days, the gRNA distribution of sorafenib-treated cells from two biologically replicated screenings was clustered separately from that of vehicle-treated cells (Additional file 1: Figure S8b). Read distributions of two biologically replicates within each condition showed a high level of correlation (Additional file 1: Figure S8c). Compared to control gRNAs, some circRNA-targeting gRNAs were significantly depleted after sorafenib selection, indicating that the corresponding genes might be sorafenib-resistant circRNAs (Fig. 4c). A subset of circRNAs, whose corresponding gRNAs were significantly depleted after sorafenib selection, were identified by MAGeCK algorithm with FDR < 0.1 (Fig. 4d, e, Additional file 10: Table S9). To test the reliability of this screening, a group of the circRNA candidates with significantly depleted gRNAs in our screening (Fig. 4d, e) were chosen for further validation. Among these circRNA candidates, approximately half of them were upregulated in sorafenib-resistant Huh7 cells according to the previously published RNA-seq data [25] (Additional file 1: Figure S8d). The circular structure of these circRNA candidates was confirmed by RNase R treatment (Additional file 1: Figure S8e). We designed BSJ site-targeting gRNAs for the circRNAs and confirmed that the majority of the tested circRNAs could be knocked down by Cas13d-mediated RNA degradation (Additional file 1: Figure S8f). Furthermore, knockdown of these circRNAs suppressed Huh7 cell proliferation only in the presence of sorafenib (Additional file 1: Figure S8g), suggesting that these circRNAs were involved in sorafenib resistance and hence validating the Cas13d screening result. Among these circRNA candidates, knockdown of circCNIH4 and circFMNL2 most significantly sensitized Huh7 cells to sorafenib treatment (Additional file 1: Figure S8g). Knockdown of these two circRNAs was achieved by another set of gRNAs (Fig. 4f, h), which effectively impeded cell proliferation in the presence of sorafenib (Fig. 4g, i). In parallel with the screening, we generated sorafenib-resistant Huh7 cells (Huh7_S cells) by consistently treating the cells with sorafenib for approximately 4 months. The cells acquired resistance to sorafenib was evidenced by increased tolerance to sorafenib treatment (Additional file 1: Figure S8h). The Huh7_S cells had elevated levels of circCNIH4 and circFMNL2 compared with the parental cells that were more sensitive to sorafenib (Fig. 4j). Importantly, knockdown of these two circRNAs in Huh7_S cells consistently sensitized the cells to sorafenib treatment (Fig. 4k, l), confirming the essential role of these two circRNA in sorafenib resistance.

Multiple pathways have been identified as underlying mechanisms of primary and acquired resistance to sorafenib, such as Ras/Raf/MEK/ERK, PI3K/AKT, and JAK-STAT signaling pathways [26, 27]. We found that although circFMNL2 knockdown did not affect the phosphorylation of ERK and STAT3 (Additional file 1: Figure S9a), it significantly inhibited the phosphorylation of AKT and the downstream S6 ribosomal protein, which were upregulated in sorafenib-treated cells (Additional file 1: Figure S9b). The inhibition effect was only observed in the sorafenib treatment group, which was consistent with the cell proliferation result demonstrating that knockdown of circFMNL2 suppressed Huh7 cell proliferation only in the presence of sorafenib (Fig. 4i, l). Taken together, these data further establish that our optimized Cas13d screening strategy is a reliable platform for identifying functional circRNAs with a much smaller false positive rate compared with shRNAs.

In addition to circRNAs, we further confirmed the ability of the CRISPR/Cas13d system to validate the function of known essential linear coding and long noncoding RNAs (lncRNAs) (Additional file 1: Figure S10). The successful validation of the functional protein-coding genes and lncRNAs demonstrates in turn the wide applicability of the CRISPR/Cas13d system in identifying the function of diverse transcripts species.

## Discussion

Different approaches have been developed to study the function of linear transcripts, whereas functional analysis on circRNAs has been challenging. The development of an appropriate tool to knock down circRNAs without affecting their cognate linear RNAs is key to understanding the functional and biological relevance of circRNAs. In this study, we optimized a strategy for designing gRNAs for the Cas13d system in order to achieve specific and efficient knockdown of circRNAs for functional studies. In general, CasRx paired with gRNAs containing 24 nt spacers could silence on-target circRNAs with high efficiencies and showed reduced silencing effects at closely matched off-target sites. The efficiency of optimized Cas13d knockdown of circRNAs was comparable to that of the widely used RNAi knockdown approach, but with substantially reduced off-target effects, making it well suited for systemic evaluation of circRNA functions. From side-by-side comparison of CRISPR/Cas13d and shRNA screenings, we found that the abilities of the two libraries to detect known essential linear genes were similar, but for circRNAs, shRNA screening yielded a much high rate of false positive phenotypes. Additionally, optimized Cas13d can validate the phenotypes of previously validated bona fide functional circRNAs. Importantly, using a Cas13d library to target a large number of human HCC-related circRNAs, we successfully identified circRNAs whose inhibition increased the therapeutic efficacy of the multikinase inhibitor, sorafenib, demonstrating the ability and reliability of Cas13d screening in identifying truly functional circRNAs.

Our Cas13d libraries contain a small proportion of gRNAs with the design of 20 + 4 or 4 + 20 nt across BSJ sites (as illustrated in Additional file 1: Figure S3f). The fact that gRNAs with 21 nt spacers showed on-target knockdown capacity to some extent (Fig. 1a,b, Additional file 1: Figure S3b) raised concern that those 20 + 4 or 4 + 20 nt BSJ-gRNAs may target the host linear counterparts of the targeted circRNAs, thus affecting the fidelity of the Cas13d screens. To address this concern, we removed all 20 + 4 or 4 + 20 nt BSJ-gRNAs from our gRNA library and re-analyzed the sorafenib selection-screen sequencing data. The 20 + 4 or 4 + 20 nt BSJ gRNAs account for 5% of our larger Cas13d library. As shown in Additional file 1: Figure S11a, compared to control gRNAs, a substantial number of circRNA-targeting gRNAs were still depleted after sorafenib selection, despite the removal of the 20 + 4 or 4 + 20 nt BSJ-gRNAs. The number of significant negatively selected circRNAs (as identified by MAGeCK algorithm with FDR < 0.1), whose corresponding gRNAs were depleted after sorafenib selection, is largely unchanged in the new analysis compared with the previous analysis (Additional file 1: Figure S11b). Moreover, the top negatively selected circRNAs that were experimentally validated in our previous analysis remain among the top circRNAs with the highest robust rank aggregation (RRA) scores in our new analysis (Additional file 1: Figure S11c). CircRNAs, which were experimentally validated in our previous analysis without the removal of the 20 + 4 or 4 + 20 nt BSJ-gRNAs (Fig. 4e), are labeled in colored dots (Additional file 1: Figure S11c). Furthermore, despite the removal of the 4 + 20 or 20 + 4 nt BSJ-gRNAs, the median log fold changes of the abundance of gRNAs targeting the same gene after sorafenib treatment highly correlate with that in the previous analysis (Additional file 1: Figure S11d), strengthening the conclusion that the 20 + 4 or 4 + 20 nt BSJ-gRNAs do not affect the validity of the screening. Importantly, the majority of the top experimentally validated hits, labeled in colored dots, correlate perfectly with each other, as shown by location of the hits on the perfect correlation red dashed line (Additional file 1: Figure S11d). Collectively, based on the above data, we conclude that the presence of the gRNAs with the design of 4 + 20 or 20 + 4 nt does not affect screening fidelity.

Although gRNAs with 20 nt spacers may show some on-target knockdown capacity [19, 28], our data suggest that the circRNA-targeting 20 + 4 or 4 + 20 nt BSJ-gRNAs that contain 20 nt complementary sequences to the host linear RNAs do not affect linear RNAs’ expression but only deplete targeted circRNAs (Additional file 1: Figure S3f). The data suggest that the presence of mismatch sequences in gRNAs can impair their ability to mediate RNA degradation. This observation is supported by the fact that one single mismatch or two consecutive double mismatches in a gRNA with a 24 nt spacer could block the knockdown effect to the targeted RNA (Fig. 1d, e). Two other independent studies also demonstrated that mismatches between gRNA spacer and target RNA impede Cas13d cleavage activity [28, 29]. Collectively, this evidence suggests that correct base pairing between gRNA spacer and target RNA is essential for target RNA cleavage. Future studies on the high-resolution crystal structure of Cas13d may shed light on the mechanism of mismatch intolerance. Nevertheless, to fully exclude the possibility that circRNA-targeting gRNAs could potentially target host linear mRNAs, gRNAs targeting the center region of BSJ sites of circRNAs, in another word having a high number of mismatch sequences when matched to host linear RNAs, are recommended for circRNA studies.

In general, our study is among the first to develop a high-throughput screening system with CRISPR-Cas13d for manipulating circRNAs and uncovering important biological function of these molecules. While several studies have already pointed to extensive expression of circRNA species in HCC, there has been limited functional studies that clearly demonstrate their biological relevance and role in the disease process. Although our initial Cas13d screening is suggestive that a group of highly expressed circRNAs in HCC may not necessarily be relevant for cell survival, an expanded screening clearly identifies circRNAs that function to promote sorafenib resistance. Indeed, chronic treatment of HCC cells with sorafenib to promote outgrowth of resistant clones was associated with upregulation of circRNAs depleted in our screening, validating the relevance of these circRNAs in promoting resistance to the drug. Thus, our studies highlight the importance of circRNAs as a mechanism in adapting to therapeutic insult in HCC, and pave the way for future studies using the system for screening functional noncoding RNAs that are more problematic to screen with the DNA-editing CRISPR-Cas9 system.

## Conclusions

In summary, we have optimized a CRISPR/Cas13d-based approach that dramatically reduces off-target background noise in both the screening and validation of truly functional circRNAs. This approach will tremendously facilitate the annotation of the functional circRNA landscape in both physiological processes and disease pathogenesis.

## Methods

### Computational pipeline for HCC circRNAs annotation

We used the CIRCexplorer2 pipeline [12] to annotate expressed circRNAs in HCC patients as candidate circRNA targets. In brief, sequencing reads of ribo-depleted total RNA-seq datasets [16] (GSE77509) of 40 samples from 20 HCC patients (each with one pair of primary tumor and adjacent normal tissue sample) were aligned to the GRCH37/hg19 human reference genome by STAR (parameters: --chimSegmentMin 10) to identify chimeric junction reads. Chimeric junction reads were then filtered and compared against the UCSC gene annotation (updated at 2016/9/17) to quantify the expression of circRNAs using CIRCexplorer2 (Additional file 2: Table S1), and 134 expressed circRNAs were selected with RPM (reads per million mapped reads) ≥ 0.1 in all the 20 HCC patients as candidate circRNAs for further screening. For further experimental validation, a subset of circRNAs were selected according to the following criteria: (a) average fold change ≥ 1.5 between primary tumors and adjacent normal tissues from 20 HCC patients, (b) conserved between human and mouse (at least two unique reads in mouse liver samples). This filtering yielded 20 circRNAs (Additional file 3: Table S2), and top 5 upregulated and top 5 downregulated circRNAs were then selected for experimental validation.

### Cell culture and treatment

Human cell lines including Huh7, HepG2, Hep3B, SK-Hep1, SNU475, SNU423, SNU387, PLC/PRF/5, HCT116, and HEK293T cells were purchased from American Type Culture Collection (ATCC). Huh7, SK-Hep1, PLC/PRF/5, HCT116, and HEK293 cells were maintained in DMEM supplemented with 10% FBS at 37 °C with 5% CO_2_. HepG2 and Hep3B cells were maintained in MEM supplemented with 10% FBS at 37 °C with 5% CO_2_. SNU475, SNU423, and SNU387 cells were maintained in RPMI 1640 supplemented with 10% FBS at 37 °C with 5% CO_2_. To generate Huh7, PLC/PRF/5, and HCT116 cells with stable expression of CasRx, the cells were transduced by EF1a-CasRx (no NLS-RfxCas13d)-2A-EGFP (modified from Addgene #109049) lentivirus, and CasRx-positive cells were then collected through cell sorting for EGFP marker. Antisense LNA GapmerRs were synthesized at QIAGEN and were transfected into Huh7 cells with Lipofectamine 2000 (Invitrogen) according to the manufacturer’s instructions with a concentration of 50 nM. Transfection of miR-7 mimic (has-miR-7, Millipore Sigma, HMI0909) was conducted using Lipofectamine 2000 (Invitrogen). For dose-gradient transfection, a total concentration of 1 nM mimic was used with varying ratios of miR-7 and negative control miRNA mimic, as indicated.

### Plasmids construction

The lentiviral gRNA and pre-gRNA-expressing backbones were constructed by cloning the human U6 promoter and CasRx gRNA or pre-gRNA scaffold (Addgene #109053, #109054) into lentiGuide-Puro (Addgene, #52963) by replacing its original U6-gRNA cassette. To construct individual gRNA or pre-gRNA-expressing vector, the annealing pairs of oligonucleotides (Invitrogen) harboring complementary sticky ends were ligated to BsmBI-cleaved gRNA or pre-gRNA backbones. The sequences of all oligonucleotides used to construct gRNA or pre-gRNA-expressing vector are shown in Additional file 11: Table S10. The oligonucleotides for shRNA were cloned into the pLKO.1-TRC vector (Addgene #10878) using AgeI/EcoRI. The sequence of all oligonucleotides used to construct shRNA-expressing vectors is shown in Additional file 11: Table S10. To construct circEGFP expressing plasmid, a partial EGFP sequence (Additional file 4: Table S3) was inserted into lentiviral backbone with two complementary sequences in the flanking intron. All the plasmids used in this study will be available from the corresponding author upon reasonable request.

### RNA isolation, qRT-PCR, RT-PCR, and northern blotting

Total RNA from cultured cells with different treatments was extracted with Trizol Reagent (Invitrogen) according to the manufacturer’s protocol. For qRT-PCR and RT-PCR, the cDNA synthesis was carried out using SuperScript IV (Invitrogen) with random hexamers. QPCR was done using SybrGreen reaction mix (Applied Biosystems) and StepOnePlus™ real-time PCR system (Applied Biosystems). The relative expression of different sets of genes was normalized to GAPDH mRNA level. Primer sequences for RT-PCR and qRT-PCR are listed in Additional file 11: Table S10. Northern blotting was carried out according to the manufacturer’s protocol (DIG Northern Starter Kit, Roche). RNA was loaded on native agarose gel or denatured PAGE gels. Digoxigenin (Dig)-labeled antisense probes were generated using T7 RNA polymerase by in vitro transcription with the RiboMAX Large Scale RNA Production System (Promega). Primer sequences for amplification of probe are listed in Additional file 11: Table S10.

### RNase R treatment

To enrich circRNA isoforms, 10 μg total RNA was diluted in 20 μl of water with 4 U RNase R/μg and 2 μl enzyme buffer (Epicenter), then incubated at 37 °C for 3 h. Ten micrograms total RNA incubated with buffer only was used as controls. Both RNase R-treated and untreated RNAs were further subjected to Trizol extraction and followed by qRT-PCR or RT-PCR.

### Nuclear/cytoplasmic RNA/protein fractionation

Cellular fractionation in Huh7 cells was performed as previously described [30]. Briefly, 2 × 10^7^ Huh7 cells were used for nuclear/cytoplasmic RNA/protein fractionation. Cell pellet was suspended by gentle pipetting in 200 μl lysis buffer (10 mM Tris pH 8.0, 140 mM NaCl, 1.5 mM MgCl2, 0.5% Igepal, 40 U/ml Recombinant RNasin Ribonuclease Inhibitor) and incubated on ice for 10 min. During the incubation, one tenth of the lysate was added to 1 ml Trizol for total RNA extraction. The rest of the lysate was centrifuged at the 1000 rpm for 3 min at 4 °C to pellet the nuclei, and the supernatant was the cytoplasmic fraction. For qRT-PCR or northern blotting, fractionated RNAs from the same amount of cells were used for cDNA synthesis or loaded into the denatured PAGE gels. For western blotting, fractionated proteins from the same amount of cells were loaded into NuPAGE Bis-Tris gel.

### Western blotting

The total protein lysate or protein lysate from different cellular fractions was extracted by RIPA buffer, then loaded into NuPAGE 4–12% gradient gels (Thermo Fisher). The protein expression of CasRx-HA was detected using anti-HA antibody (HA-tag (F7), Santa Cruz Biotechnology, sc7392). Primary antibody anti-GAPDH (Abcam, ab9485) and anti-Histone H3 (Cell Signaling Technology, 9715) were used to detect cellular fractionation markers: GAPDH and Histone H3.

### Cell proliferation assay

Cell proliferation was measured using crystal violet staining or CCK-8 kit. For crystal violet staining, cells were seeded at a concentration of 4 × 10^4^ cells (Huh7 and PLC/PRF/5 cells) or 3 × 10^4^ cells (HCT116 cells) per well in a 12-well plate and cultured for 6 days in complete medium (DMEM plus 10% FBS) at 37 °C. Cells were fixed with 10% formalin at indicated days and stained with 0.1% crystal violet. Crystal violet was then solubilized with 10% acetic acid, and their absorbance was measured using SpectraMax iD3 Multi-Mode Microplate Readers. For CCK-8 assay (Abcam, ab228554), 3 × 10^3^ cells were seeded in 96-well plates and cultured for 6 days in complete medium (DMEM plus 10% FBS) at 37 °C. At indicated time points, cells were incubated with 10 μl of CCK-8 assay solution in each well for 2 h at 37 °C. The absorbance values at 460 nm were then measured using SpectraMax iD3 Multi-Mode Microplate Readers.

### Drug treatment

Sorafenib (Cat No.: S7397) were purchased from Selleck Chemicals and dissolved in DMSO. To assess cell proliferation under the drug treatment, the cells in each condition were seeded at 4 × 10^4^ cells/well in a 12-well plate, 4 × 10^4^ cells/well in a 6-well plate, or 1 × 10^4^ cells/well in a 12-well plate and cultured for 6, 8, or 10 days accordingly in a 37 °C humidified CO_2_ incubator with the drug or vehicles containing medium refreshed every other day. The number of cells at day 6, 8, or 10 was determined by crystal violet staining, and their relative absorbance was normalized to the non-targeting control of the same day. To generate sorafenib-resistant cells, Huh7 cells were treated with either DMSO (vehicle) or 2.5 μM sorafenib (Selleck Chemicals, S7397) for approximately 4 months. Medium was changed every 3 days.

### Lentivirus preparation and transduction

Low passage HEK293T cells were transfected with Lipofectamine 2000 (Thermo Fisher Scientific) and Cas13d plasmid or guide RNA-expressing plasmid plus pMD2.G and psPAX2 packaging plasmids. After 24 h, the medium was changed to prewarmed DMEM medium. Viral supernatant was harvested 48 h later, and cellular debris was filtered out using Millipore’s 0.45 μm PVDF filter. To assess the knockdown ability of individual gRNA or shRNA, Huh7 cells stably expressing Cas13d or naive Huh7 cells were infected with gRNA or shRNA lentivirus. After 24 h post-transduction, the medium was changed to fresh medium with 2 μg/ml puromycin. After 5 days post-transduction, total RNAs were harvested for further analysis.

### RNA sequencing and analysis

For specificity analysis, RNA sequencing was performed on rRNA-depleted total RNA from cells with Cas13d and shRNA-mediated circEGFP knockdown. Total RNA was extracted from cells infected with lentiviruses carrying knockdown constructs using Trizol. rRNA-depleted total RNA-Seq libraries were prepared by the Molecular Biology Core Facilities (MBCF) at Dana-Farber Cancer institute (DFCI). RNA-Seq libraries were sequenced on an Illumina NextSeq instrument with at least 10M reads per library. RNA-Seq reads were aligned and quantified with Salmon (v0.13.1) [31] using default parameters for paired-end reads with --validateMappings flag. Human reference transcriptome available in Ensemble portal (ftp://ftp.ensembl.org/pub/release-95/fasta/homo_sapiens/cdna/) were indexed for Salmon alignment and quantification. Transcript per million (TPM) values, averaged from biological replicates, were transformed to log scale for expression correlation. To find differentially expressed genes, raw transcript counts generated with Salmon were imported into DESeq2 (v1.26.0) [32] for count normalization and differential expression analysis. Genes with no read count in at least 1 sample were not included in the analysis. Only genes that had a log2 differential expression greater than 0.5 or less than − 0.5 and a false discovery rate < 0.68 were reported to be significantly differentially expressed.

### shRNA and gRNA library design

To perform functional screening, 134 highly expressed circRNAs in HCC were selected. For each circRNA candidate, all the possible 21 nt shRNA target sequences were extracted from the back-splice junction sequence (40-nt long with 20 nucleotides at each side of back-spliced exons) and scored by siDirect version 2.0 (http://sidirect2.rnai.jp/design.cgi) and GPP web portal (https://portals.broadinstitute.org/gpp/public/). To remove possible off-target sequences, all shRNA candidates were aligned back to the human transcriptome (GENCODE V19) permitting 3 mismatches with bowtie (parameters: -n 3 -l 5 --norc -y -a). shRNA candidates with ≤ 3 mismatches were considered off-target shRNAs and were excluded from the library. The shRNA sequences were selected based on the following criteria: high on-target sequence score, high coverage of the BSJ site, high complexity of the library. The final shRNA library contained 646 shRNAs targeting 132 circRNAs, and most circRNAs had 5 shRNAs (for two circRNAs, none of the shRNAs passed the filters). To generate a comparable gRNA library, gRNA sequences were designed by extending each shRNA from 21 nucleotides to 24 nucleotides and filtered by off-target blast. To evaluate the efficiency of our screens, cell-essential genes (CRISPR score < − 1 and adjusted *p* value < 0.05 in all examined cell lines) were downloaded from a CRISPR/Cas9-based genome-wide negative selection screening study [20], and ten top cell-essential genes with the lowest mean CRISPR scores were selected as positive controls. For each positive control gene, five top shRNAs with highest adjusted score were downloaded from the Genetic Perturbation Platform (https://portals.broadinstitute.org/gpp/public/). To minimize off-target effects, all control shRNAs were aligned back to the human transcriptome (GENCODE V19) permitting 3 mismatches using bowtie (parameters: -n 3 -l 5 --norc -y -a). Corresponding gRNAs targeting positive essential genes were designed by extending shRNA sequences to 24 nt oligonucleotides. In addition, 150 random intergenic regions (RefSeq gene annotations updated at 2017/5/28) in the fly genome (dm6) were selected as negative controls. For construction of the library for 2543 circRNAs, CIRCexplore2 was used to identify expressed circRNAs in human HCC patients (RNA-seq datasets [16], GSE77509). Multiple gRNAs with 24 nt spacers across the BSJ sites in incremental steps were designed to target each of the 2543 circRNAs. To remove possible off-target sequences, all gRNA candidates were aligned back to the human transcriptome (GENCODE V19), permitting 3 mismatches with bowtie (parameters: -n 3 -l 5 --norc -y -a). gRNA candidates with ≤ 3 mismatches were considered off-target gRNAs and were excluded from the library.

### Construction of the Cas13d gRNA and shRNA libraries and libraries screening

Cas13d gRNA library were synthesized as 94-mer oligonucleotides (CustomArray), caccgaacccctaccaactggtcggggtttgaaacNNNNNNNNNNNNNNNNNNNNNNNNttttttaagcttggcgtaactagatcttgagacaa (N indicates the 24 nt spacer sequence), and amplified by PCR as a pool using the following primers: tatatatcttgtggaaaggacgaaacaccgaacccctaccaactggtcggggtttgaaac (Forward), cttttaaaattgtggatgaatactgccatttgtctcaagatctagttacgccaagc (Reverse). shRNA library were synthesized as 92-mer oligonucleotides (CustomArray),

ggaaaggacgaaacaccggNNNNNNNNNNNNNNNNNNNNNctcgagNNNNNNNNNNNNNNNNNNNNNtttttgaattctcgacctcgagaca (N indicated 21 nt target sequence), and amplified by PCR as a pool using the following primers: taacttgaaagtatttcgatttcttggctttatatatcttgtggaaaggacgaaacaccgg (Forward), cccccttttcttttaaaattgtggatgaatactgccatttgtctcgaggtcgagaattc (Reverse). The PCR product was purified and then cloned into gRNA-expressing or shRNA-expressing vector using NEBuilder HiFi DNA Assembly Master Mix (NEB #E2621). The 100 ng product was then transformed into Endura ElectroCompetent cells according to the manufacturer’s directions. Clones were scraped off the LB plates and plasmid DNA was extracted using PureLink™ HiPure Plasmid Maxiprep Kit (Thermo Fisher, K210007). The libraries were submitted for next-generation sequencing to confirm the coverage and diversity of gRNA and shRNA libraries. The lentivirus of gRNA or shRNA library was produced by co-transfection of library plasmids with two viral packaging plasmids psPAX2 and pMD2.G into HEK293 cells using Lipofactamine 2000 (Invitrogen). Huh7 cells were transduced with lentivirus libraries at multiplicity of infection (MOI) ~ 0.3. Replicated transductions were performed. Twenty-four hours after transduction, cells were cultured with fresh medium containing 2 μg/ml puromycin. After 2 days of puromycin selection, genomic DNA was extracted as day 0. For cell growth screening, cells were passaged every 3 days and maintained a coverage of > 500 cells per gRNA or shRNA. For drug selection screening, cells were treated with DMSO (vehicle) or 2.5 μM sorafenib, and medium was changed every other day. After 14 days of screening, genomic DNA was extracted for replicated samples. gRNA and shRNA inserts were amplified using 10 different NGS-lib-Forward primers paired with Reverse primers containing unique barcode (Additional file 11: Table S10). gRNA and shRNA distribution were determined by next-generation sequencing. The libraries were sequenced on the Illumina MiSeq or Hiseq according to the user manual (Harvard Medical School Biopolymers Facility, Boston).

### Computational analysis of screens

The screening sequencing data was analyzed using MAGeCK (v0.5.8) [33]. MAGeCK “count” command was used to generate read counts of all samples as previously described [34]. Briefly, raw read counts were normalized with DESeq2 then rlog transformed for generation of correlation heatmaps and PCA plots (Fig. 3c, Additional file 1: Figures S4b, S6a,b and S8b, c). MAGeCK “test” command was used to identify the top negatively and positively selected circRNAs as previously described [34]. MAGeCK estimates the level of negative (or positive) selection of each circRNA by comparing the rankings of all gRNAs or shRNAs targeting that circRNA with a null model, where all gRNAs/shRNAs are distributed uniformly in the ranked list. The α-Robust Rank Aggregation (α-RRA) algorithm was used to calculate the “RRA score” of each circRNA to describe the degree of negative (or positive) selection. The *P* value of the RRA score was computed by permuting all circRNAs, and adjusted for multiple comparison correction with the Benjamini-Hochberg method. A detailed description of the algorithm is reported in the original study [33].

### Gene set enrichment analysis

Preranked GSEA of gRNAs and shRNAs for positive controls (known essential genes) was conducted using the fgsea (v1.12.0) R package.

## Supplementary Information

**Additional file 1:** **Supplementary Figures.** CRISPR/Cas13d system is an effective approach to study the function of circRNAs in a high-throughput manner.

**Additional file 2: Table S1.** List of circRNAs in 20 HCC patients.

**Additional file 3: Table S2.** List of 134 highly expressed HCC circRNAs with 20 conversed circRNAs labeled in yellow.

**Additional file 4: Table S3.** Partial EGFP sequence used for construction of the circEGFP plasmid.

**Additional file 5: Table S4.** List of oligonucleotides in the Cas13d library.

**Additional file 6: Table S5.** List of oligonucleotides in the shRNA library.

**Additional file 7: Table S6.** MAGeCK results of negatively and positively selected genes for shRNA screening.

**Additional file 8: Table S7.** MAGeCK results of negatively and positively selected genes for Cas13d screening.

**Additional file 9: Table S8.** List of oligonucleotides in the large Cas13d library for drug selection screening.

**Additional file 10: Table S9.** MAGeCK results of negatively and positively selected genes for drug selection screening.

**Additional file 11: Table S10.** All primer sequences used in the study.

**Additional file 12.** Northern and Western blots. Full, uncut blots.

## Data Availability

The sequencing datasets generated and analyzed in the current study are available in the Gene Expression Omnibus (GEO) database with accession number GSE162720 [35].
