# Supplementary material for: Optimized RNA-targeting CRISPR/Cas13d technology outperforms shRNA in identifying functional circRNAs
Source: Genome Biol. 2021 Jan 21;22:41. doi: 10.1186/s13059-021-02263-9 (PMC7818937; doi:10.1186/s13059-021-02263-9)
Supplement: Supplementary file 1 — Additional file 1: Supplementary Figures. CRISPR/Cas13d system is an effective approach to study the function of circRNAs in a high-throughput manner. [file 13059_2021_2263_MOESM1_ESM.docx]

**Inventory of Supplementary Figures**

Fig. S1 is related to main Fig. 1.

Fig. S2 is related to main Fig. 1.

Fig. S3 is related to main Fig. 1 and 2.

Fig. S4 is related to main Fig. 3.

Fig. S5 is related to main Fig. 3.

Fig. S6 is related to main Fig .3.

Fig. S7 is related to main Fig. 3.

Fig. S8 is related to main Fig. 4.

Fig. S9 is related to main Fig. 4.

Fig. S10 is related to main Fig. 4.

Fig. S11 is related to main Fig. 4.

**Supplementary Figures and Figure Legends**

**
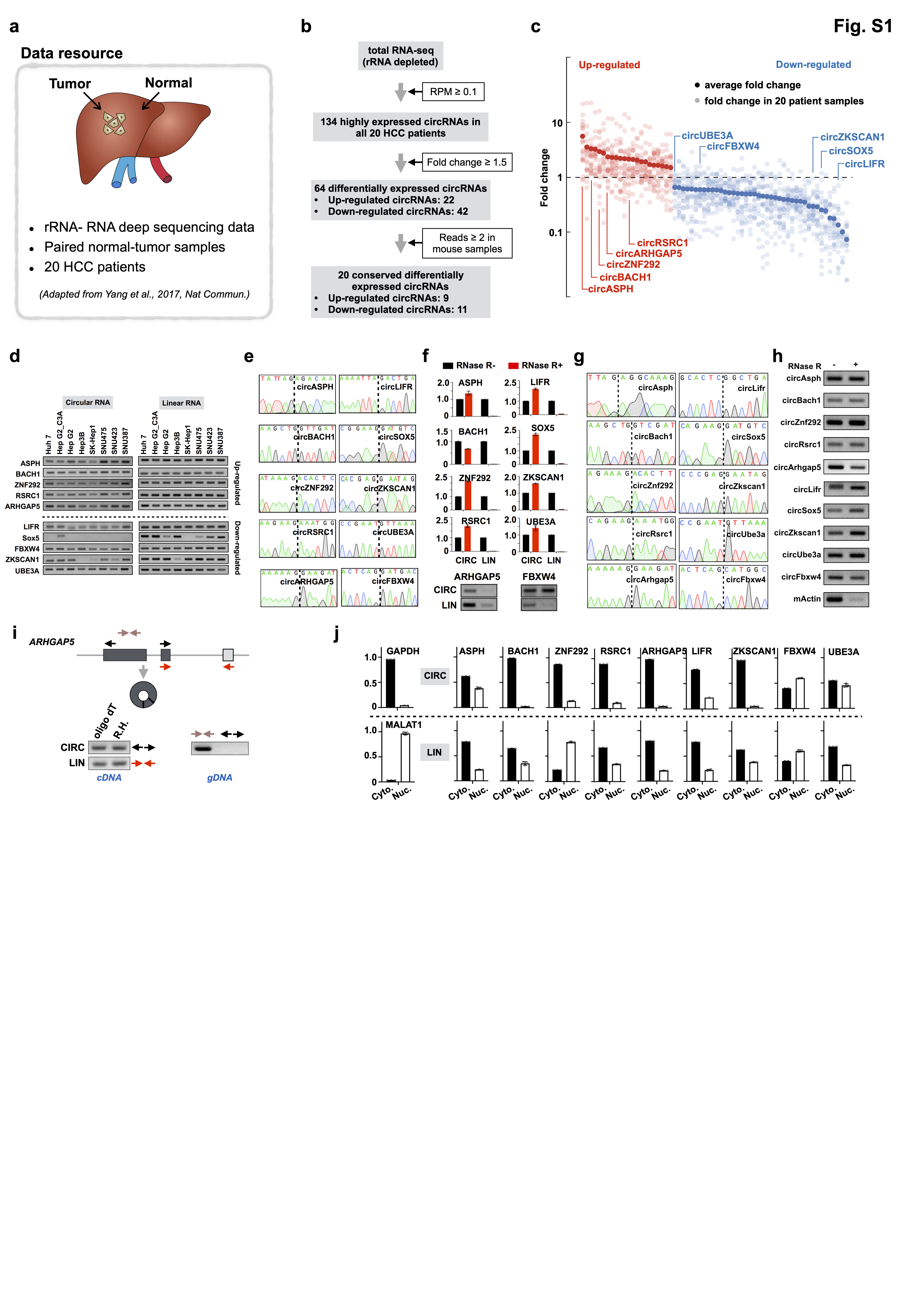
**

**Fig. S1 | Identification and characterization of circRNAs in HCC. (a)**

Schematic drawing of data resource used for identification of circRNAs in HCC. **(b)** The schematic diagram shows the computational pipeline for the systematic identification of circRNAs in samples from HCC patients. **(c)** Scatterplots showing fold change of differentially expressed circRNAs in samples from HCC patients. X axis, 64 circRNAs were rank-ordered by differential expression between primary tumor samples and paired-adjacent normal tissues. Light colored dots represent the fold change of circRNAs in each paired sample from 20 HCC patients, and dark colored dots represents the average fold change. Y axis, fold change of the expression level of individual circRNAs. A subset of circRNAs validated herein are labeled. **(d-e)** Expression of human circRNAs were validated by RT-PCR (d), followed by Sanger sequencing (e). RT-PCR validation of 10 conserved differentially expressed circRNAs in 8 human HCC cell lines: circRNAs and their corresponding linear transcripts were amplified with divergent and convergent primers. Left panel, agarose electropherogram of circRNA PCR products. Right panel, agarose electropherogram of the corresponding linear mRNA PCR products (d). PCR products were subjected to Sanger sequencing, back-splicing junction sites are indicated by dash lines (e). **(f)** RNase R validation of 10 selected circRNAs. circRNAs together with the corresponding linear mRNAs were amplified by qRT-PCR or RT-PCR from cDNA prepared from RNA non-treated or treated with RNase R. **(g-h)** Validation of 10 conserved circRNAs in mouse liver samples. BSJ sites were indicated by dashed lines (g). circRNAs were amplified by RT-PCR from mouse liver cDNA prepared from non-treated or treated with RNase R. Mouse actin mRNA was used as a negative control (h). **(i)** Characterization of “circ”ARHGAP5. Top, schematic view of ARHGAP5 genomic locus. Bottom left, “circ”ARHGAP5 and its corresponding linear transcripts were amplified with divergent (black arrows) and convergent primers (red arrows) using the templates from oligo-dT Reverse Transcription. Bottom right, “circ”ARHGAP5 could not be amplified from genomic DNA with divergent primers (black arrows). The convergent primers (brown arrows) were used as a RT-PCR control. **(j)** Subcellular localization of human circRNAs. Bar plots represent relative abundance of RNAs in nuclear and cytoplasmic fractions. The relative distribution of GAPDH and MALAT1 transcripts, predominantly localized to the cytoplasm and nucleus, respectively, confirmed a successful cellular fractionation. Error bars in **f**, **j** indicating the mean ± s.d. of three technical replicates.

**
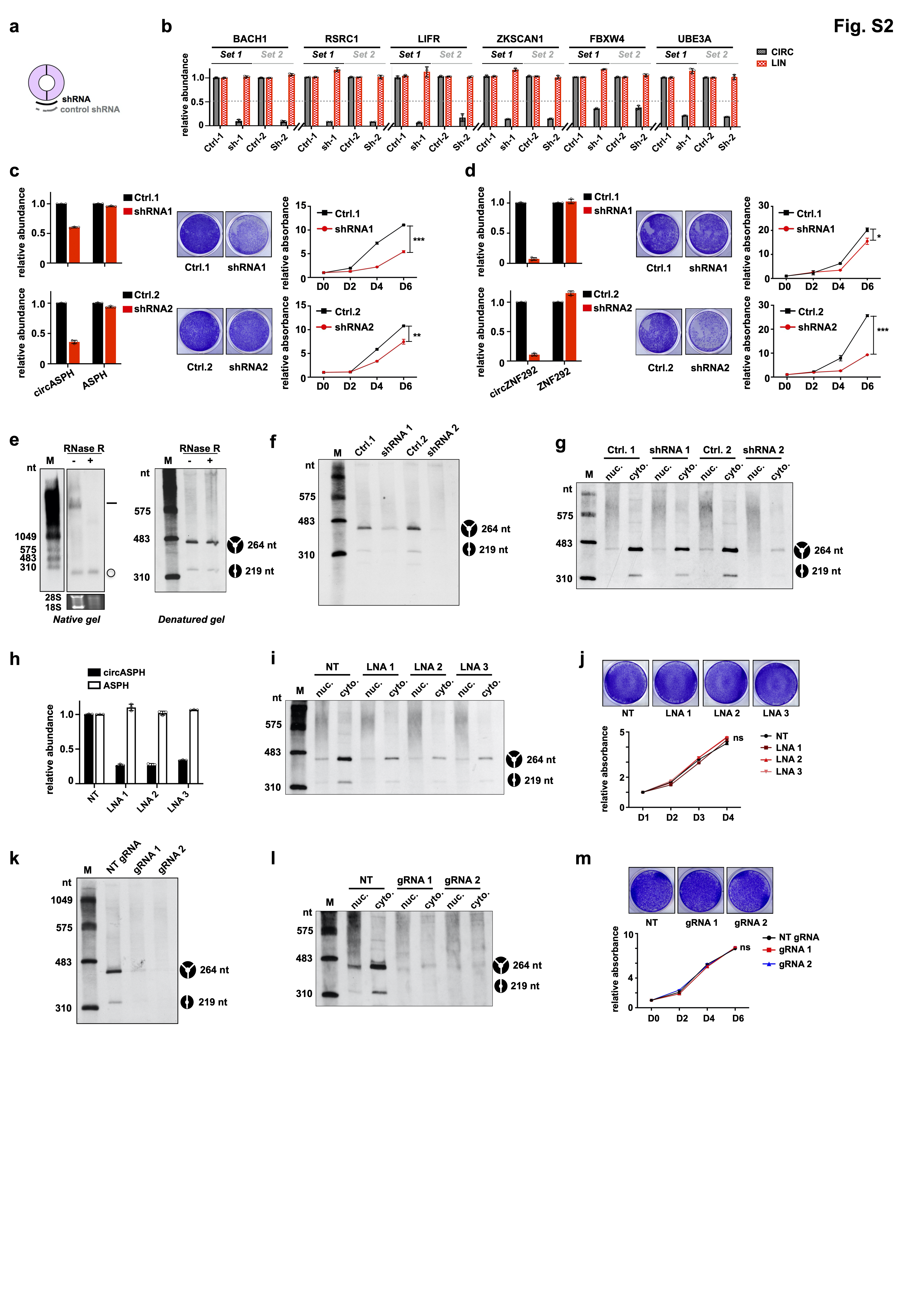
**

**Fig. S2** **| Targeting conserved HCC circRNAs with shRNAs.** **(a)** Schematic drawing of the strategy to silence the expression of circRNAs with shRNAs targeting the BSJ sites. ShRNAs with half-scrambled sequence were used as controls. **(b)** Relative expression levels of circRNAs and their parental genes upon knock-down of circRNAs by two sets of shRNAs in Huh7 cells. **(c)** Knockdown of circASPH in Huh7 cells. Relative expression levels of circASPH and its parental mRNA upon knock-down of circASPH by two sets of shRNAs in human Huh7 cells (left panel). Proliferation rates of control and circASPH-silenced Huh7 cells (right panel). **(d)** Knockdown of circZNF292 in Huh7 cells. Relative expression levels of circZNF292 and its parental mRNA upon knock-down of circZNF292 by two sets of shRNAs in human Huh7 cells (left panel). Proliferation rates of control and circZNF292-silenced Huh7 cells (right panel). **(e)** Northern Blot (NB) showing the RNase R resistance of circASPH. 10 μg total RNAs were treated with or without RNase R, and then used for NB on either native agarose gel (left) or denatured PAGE (right) for circASPH. Note that circASPH contains an alternative internal exon, which leads to the production of two isoforms of circASPH sharing the same BSJ site. Since the two isoforms of circASPH only have 45 nt difference in size, the resolution of native agarose gel is not enough to separate these two isoforms. Due to the separation range of PAGE, the larger ASPH mRNA could not be detected in denatured PAGE northern blot. **(f)** Denatured NB showing the relative abundance of two isoforms of circASPH after knockdown of circASPH with two shRNAs. **(g)** Denatured NB showing knockdown of both nuclear and cytosolic circASPH by shRNA-mediated degradation. **(h)** Relative expression levels of circASPH and its parental mRNA upon knockdown of circASPH by three LNAs in Huh7 cells. LNA, locked nucleic acid. **(i)** Denatured NB showing knockdown of both nuclear and cytosolic circASPH by LNA-mediated degradation. **(j)** Proliferation rates of control and LNA-mediated circASPH-knockdown Huh7 cells. The number of cells was detected by staining with crystal violet, and representative pictures are shown on the top, while the proliferation curves are shown on the bottom. **(k)** Denatured NB showing knockdown of circASPH upon knock-down of circASPH by two gRNAs in Huh7 cells. **(l)** Denatured NB showing knockdown of both nuclear and cytosolic circASPH by Cas13d-mediated degradation. **(m)** Proliferation rates of control and Cas13d-mediated circASPH-knockdown Huh7 cells. The number of cells was detected by staining with crystal violet, and representative pictures are shown on the top, while the proliferation curves are shown on the bottom. The data shown are from one of two biological replicates with similar results, and error bars indicating the mean ± s.d. of three technical replicates. **p* < 0.05, ***p* < 0.01, ****p* < 0.001 (unpaired student’s *t* test). ns, not significant.

**
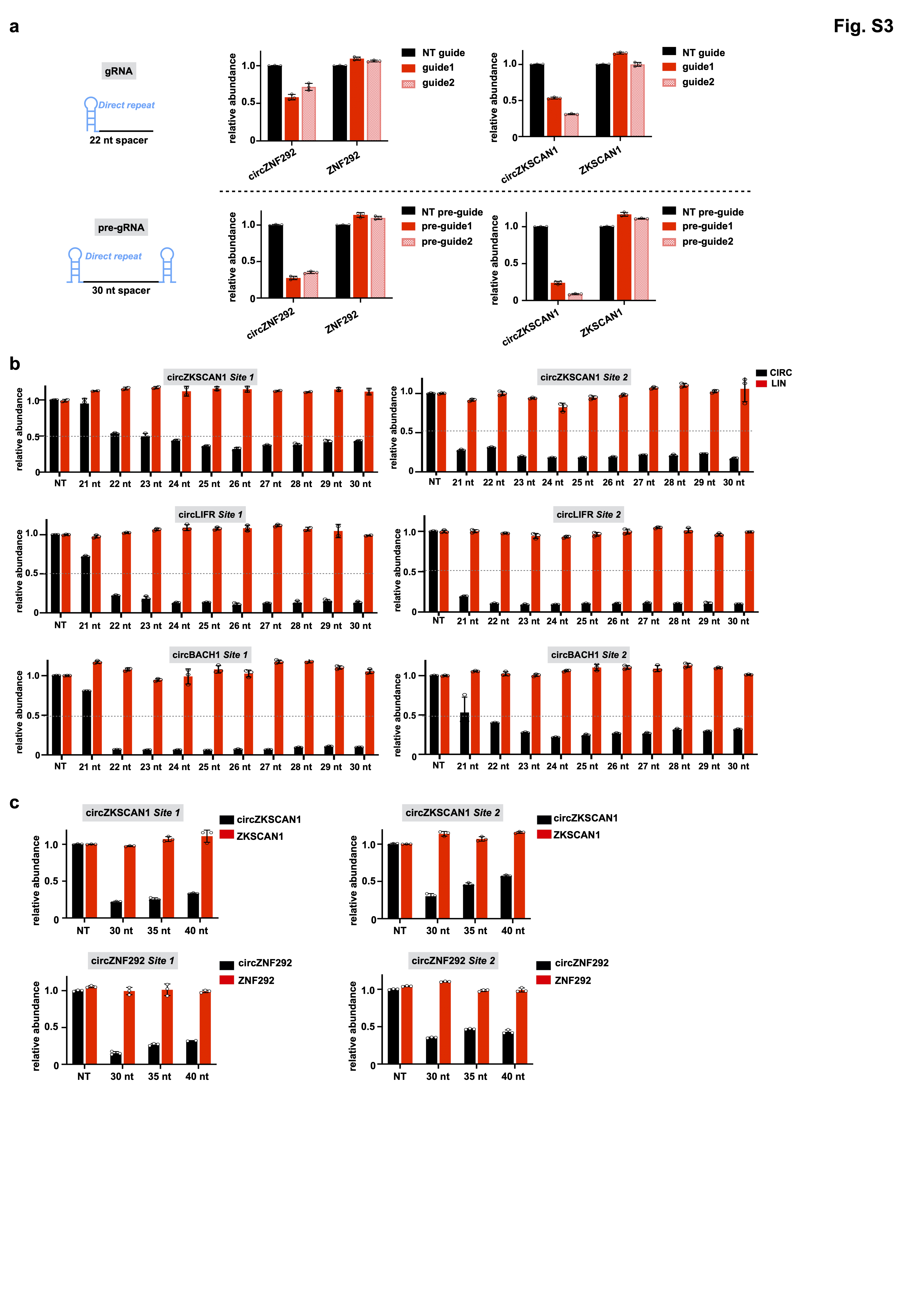
**

**
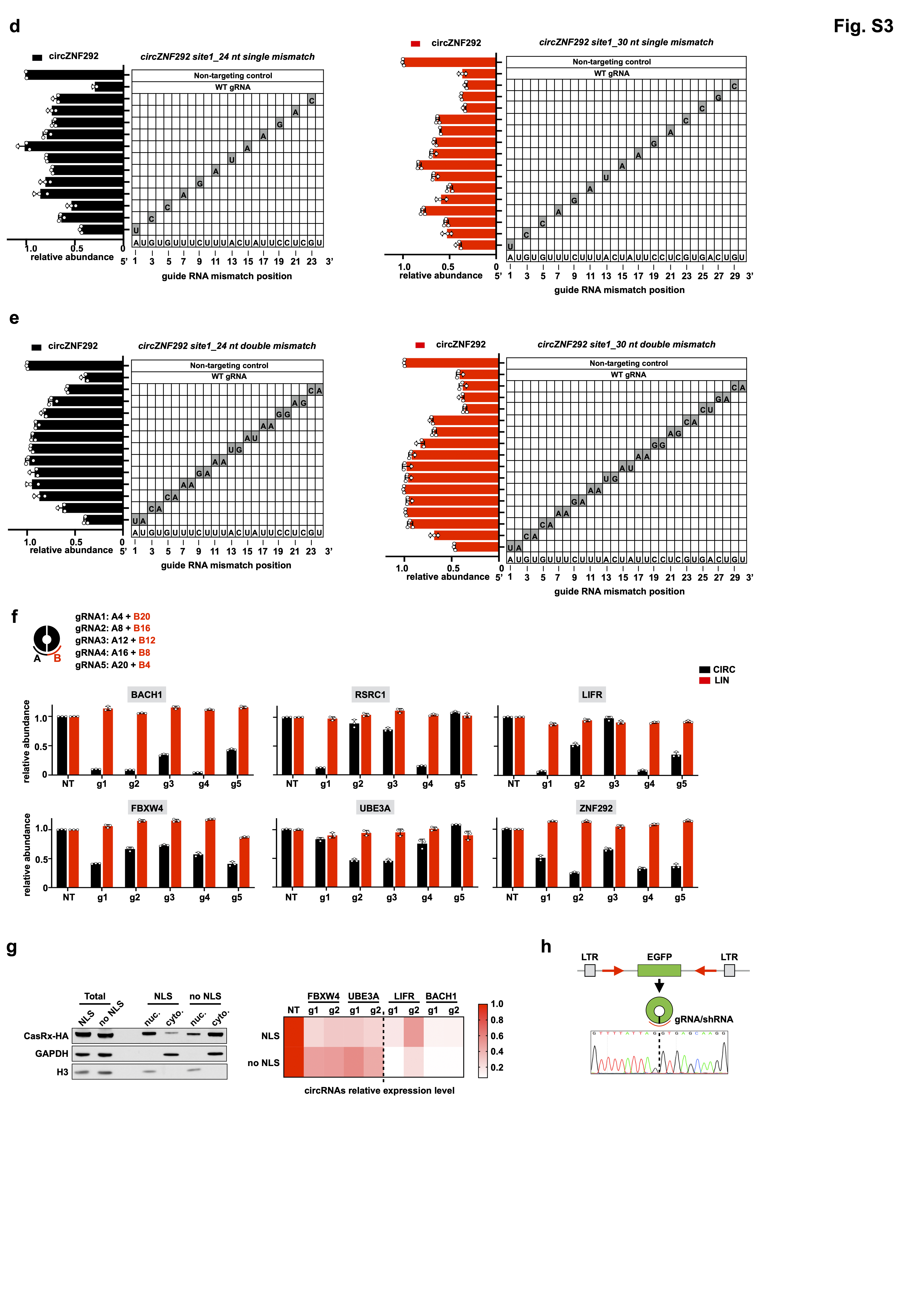
**

**Fig. S3 | Adaptation of CRISPR-Cas13d system to silence circRNAs. (a)** Comparison of gRNA and pre-gRNA for knockdown of endogenous circRNAs reveals pre-gRNA to be the more effective guide RNA architecture. Schematic for constructs expressing guide RNAs. pre-gRNA, unprocessed guide RNA containing a single 30 nt spacer sequence flanked by 2 full-length 36 nt Direct repeats. gRNA, predicted mature guide RNA with a single 30 nt processed Direct repeat and 22 nt spacer sequence. NT, non-targeting. **(b)** Bar plots showing the relative expression of three more circRNAs and their linear parental RNAs upon knockdown of circRNAs with different length of gRNAs targeting two regions of BSJ site of each circRNA. NT, non-targeting. **(c)** Bar plots showing the relative expression of circZKSCAN1 and its parental linear mRNA (top) or circZNF292 and its parental linear mRNA (bottom) upon knockdown of circRNAs with gRNAs containing 30 nt, 35 nt and 40 nt length spacers. **(d)** Knockdown of circZNF292 evaluated with gRNAs containing 24 nt length spacer (left) or 30 nt length spacer (right) with single mismatch at varying positions across the spacer sequence. The gray boxes in the grids show the position of Watson-Crick transversion mismatches. The wild-type sequence is shown at the bottom of each grid. **(e)** Knockdown of circZNF292 evaluated with gRNAs containing 24 nt length spacer (left) or 30 nt length spacer (right) with consecutive double mismatch at varying positions across the spacer sequence. The gray boxes in the grids show the position of Watson-Crick transversion mismatches. The wild-type sequence is shown at the bottom of each grid. **(f)** Top, schematic view of BSJ-site targeting gRNA design. Five gRNAs with fixed 24 nt spacers across the BSJ sites in incremental steps were designed to target each circRNA. Bottom, bar plots showing the relative expression of six circRNAs and their linear parental RNAs upon knockdown of circRNAs with gRNAs targeting different regions of BSJ site of each circRNA. NT, non-targeting. **(g)** Comparison of CasRx with or without NLS for knockdown of circRNAs with different cellular localization. Left, western blot showing the cellular localization of CasRx with or without NLS. GAPDH and Histone H3 were used as cellular fractionation markers for nucleus and cytoplasm, respectively. Right, heatmap display of relative knockdown level of circRNAs with nuclear distribution (circFBXW4 and circUBE3A) and cytosolic (circLIFR and circBACH1) circRNAs. NLS, nuclear localization signal. g1 and g2 represent two independent gRNAs. **(h)** Schematic drawing of circular EGFP expression vector (Top). Partial sequence of EGFP was inserted into circRNA expression vector with fully complementary sequences in the flanking introns to facilitate the biogenesis of circEGFP. The complementary sequences are indicated with red arrow to show the polarity. Bottom, Sanger sequencing result confirms the BSJ site of circEGFP, as indicated by black dash line. The data shown are from one of two biological replicates with similar results, and error bars indicating the mean ± s.d. of three technical replicates.

**
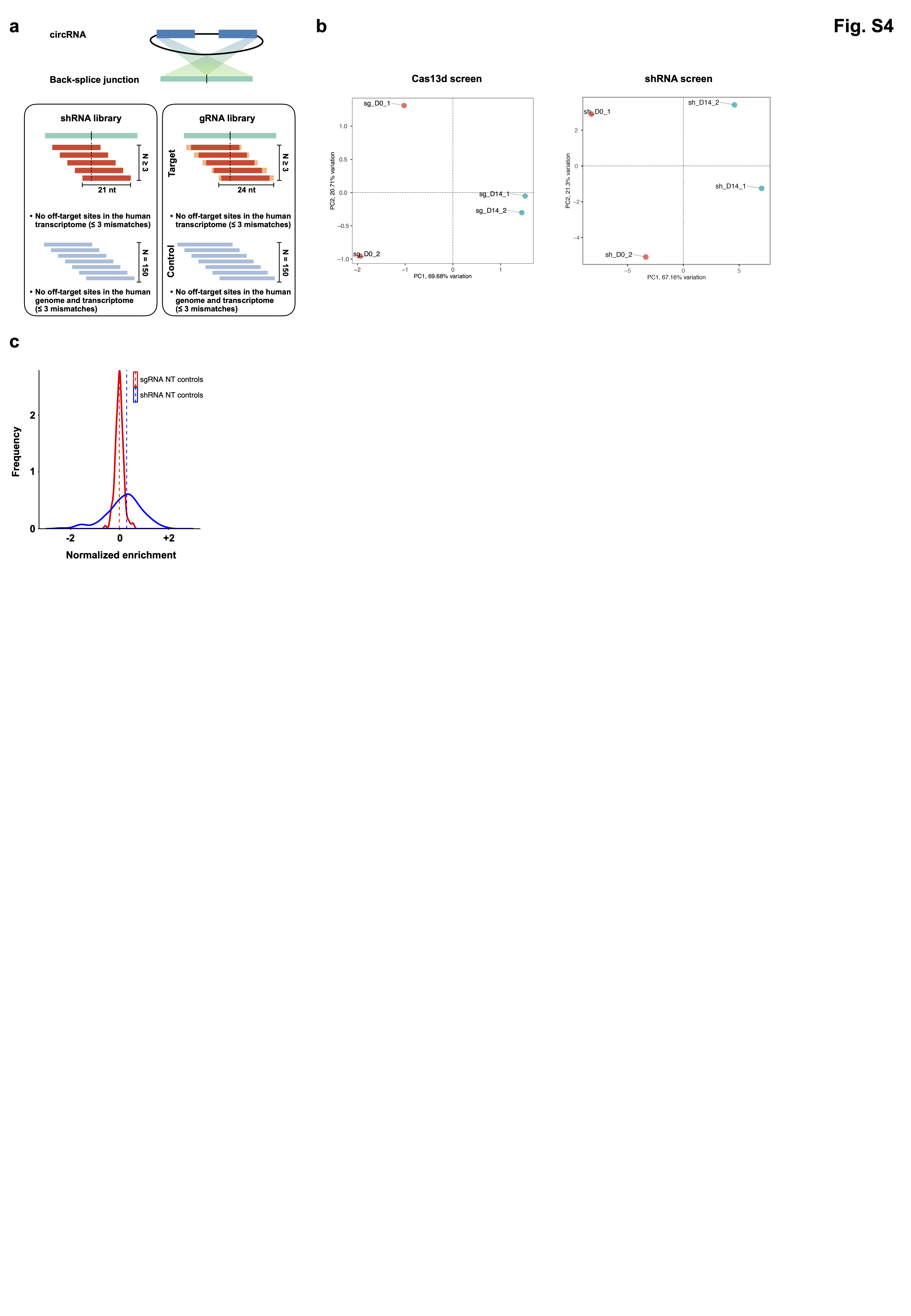
**

**Fig. S4 | Design of Cas13d and shRNA libraries. (a)** Schematic drawing of shRNA and gRNA libraries (see details in Methods). Position-matched shRNAs and gRNAs were designed to target the BSJ sites of 134 circRNAs. shRNAs and gRNAs targeting mRNAs of 10 essential genes serve as positive controls. 150 non-human genomic sequence targeting shRNAs and gRNAs are included as negative controls. (**b**) Principal component analysis of gRNA (left) or shRNA (right) levels across the four generated sequencing libraries. PC, principal component. **(c)** Histograms representing the relative distribution of non-targeting control gRNAs and shRNAs.

**
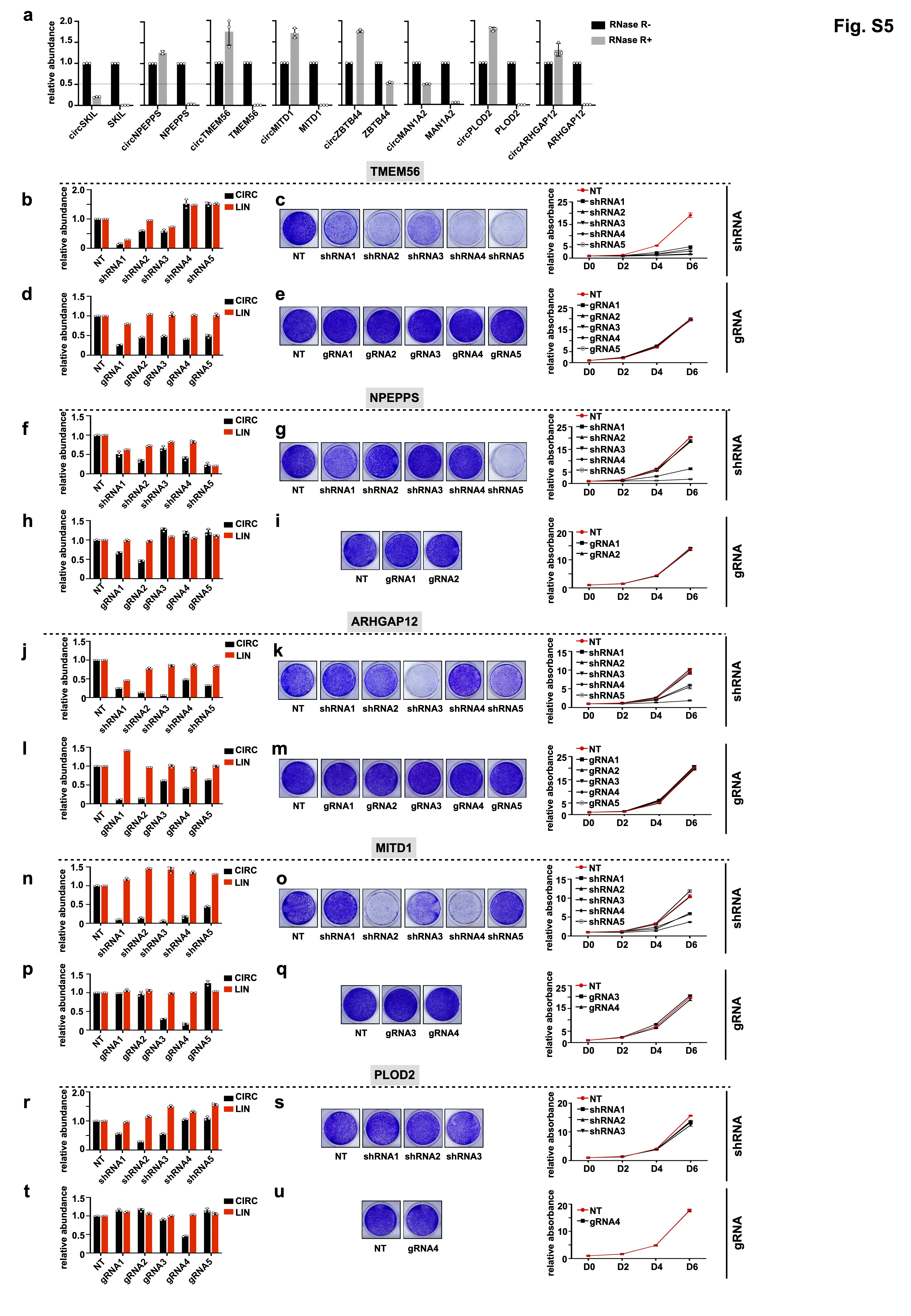
**

**Fig. S5 | High false positive rate of shRNA functional screen for circRNAs. (a)** RNase R validation of 8 selected circRNAs. circRNAs together with the corresponding linear mRNAs were amplified by qRT-PCR from cDNA prepared from RNA non-treated or treated with RNase R. **(b, d)** Relative expression levels of circTMEM56 and its parental mRNA upon knock-down of circTMEM56 by shRNAs (b) and gRNAs (d) in Huh7 cells. **(c, e)** Proliferation rates of control and shRNA-mediated (c) and Cas13d-mediated (e) circTMEM56-silenced Huh7 cells. The number of cells was detected upon staining with crystal violet, and representative pictures are shown on the left, while the proliferation curves are shown on the right. **(f, h, j, l, n, p, r, t)** same as in (b, d) for circNPEPPS, circARHGAP12, circMITD1 and circPLOD2. **(g, i, k, m, o, q, s, u)** same as in (c, e) for circNPEPPS, circARHGAP12, circMITD1 and circPLOD2. The data shown are from one of two biological replicates with similar results, and error bars indicating the mean ± s.d. of three technical replicates.


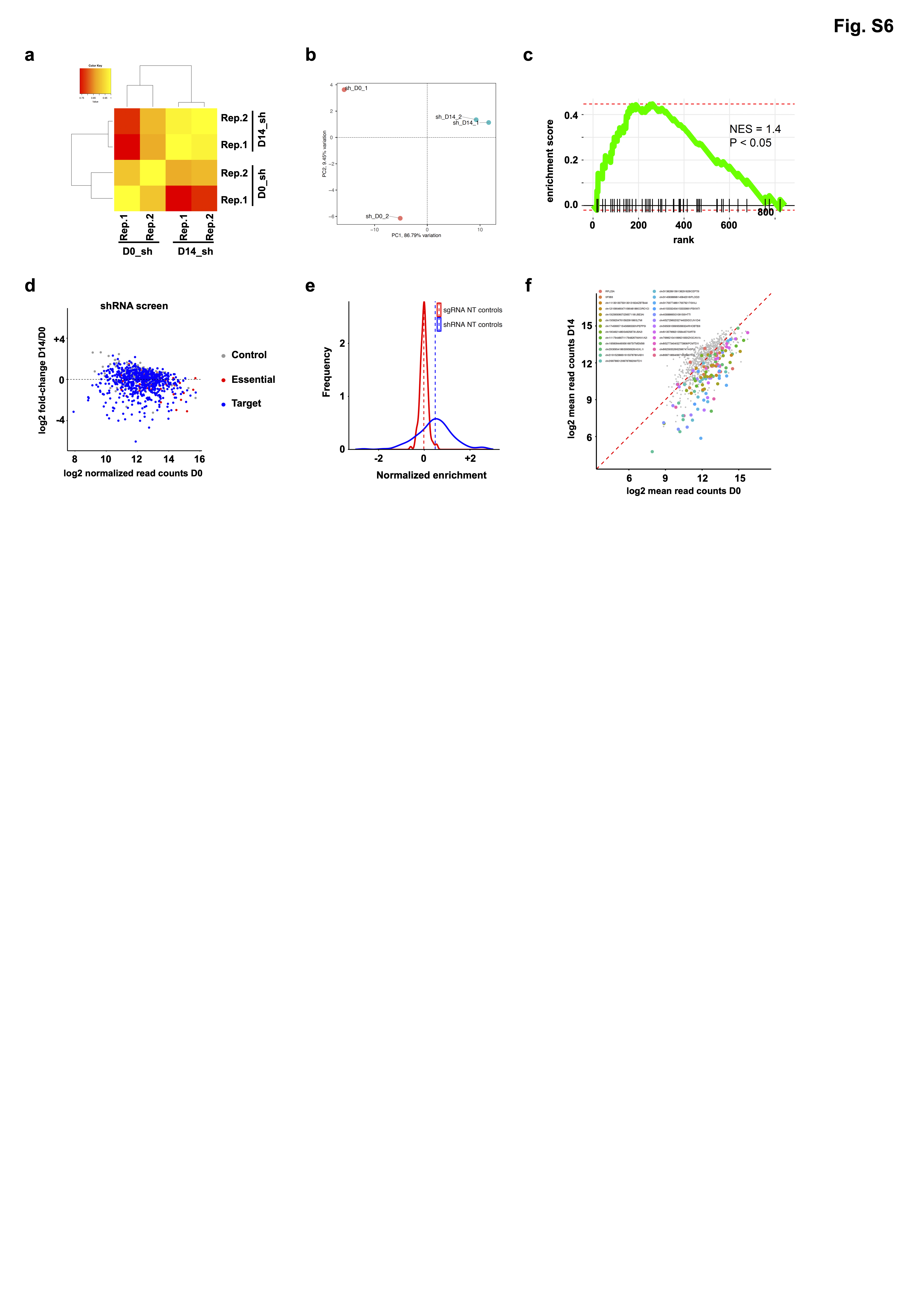


**Fig. S6 | Identification of negatively selected circRNAs from the second shRNA screen. (a)** Correlation heatmap showing the Pearson correlation coefficient between the levels of shRNAs in biological replicates of day-0 samples (D0) and day-14 enrichment samples (D14). **(b)** Principal component analysis of shRNA levels across the four generated sequencing libraries. PC, principal component. **(c)** Gene set enrichment analysis (GSEA) revealed that shRNAs targeting known essential genes are significantly enriched in negative selections by the second shRNA screen. The known essential genes serve as positive controls. The degree of enrichment is measured as normalized enrichment score (NES). **(d)** Scatterplot showing fold-change of shRNA normalized read counts in D14 vs. D0. Control, non-targeting controls; Essential, positive controls targeting known essential genes; Target, circRNAs highly expressed in HCC. **(e)** Histograms representing the relative distribution of non-targeting control gRNAs and shRNAs in the second shRNA screen. **(f)** Scatterplots showing negatively selected shRNAs and corresponding genes from the second shRNA screen with FDR < 0.25. CircRNAs are indicated with genomic locations and the host gene name at the end (e.g. chr11|130130750|130131824|ZBTB44|). Positive controls only have gene names without genomic location (e.g. RPL23A).

**
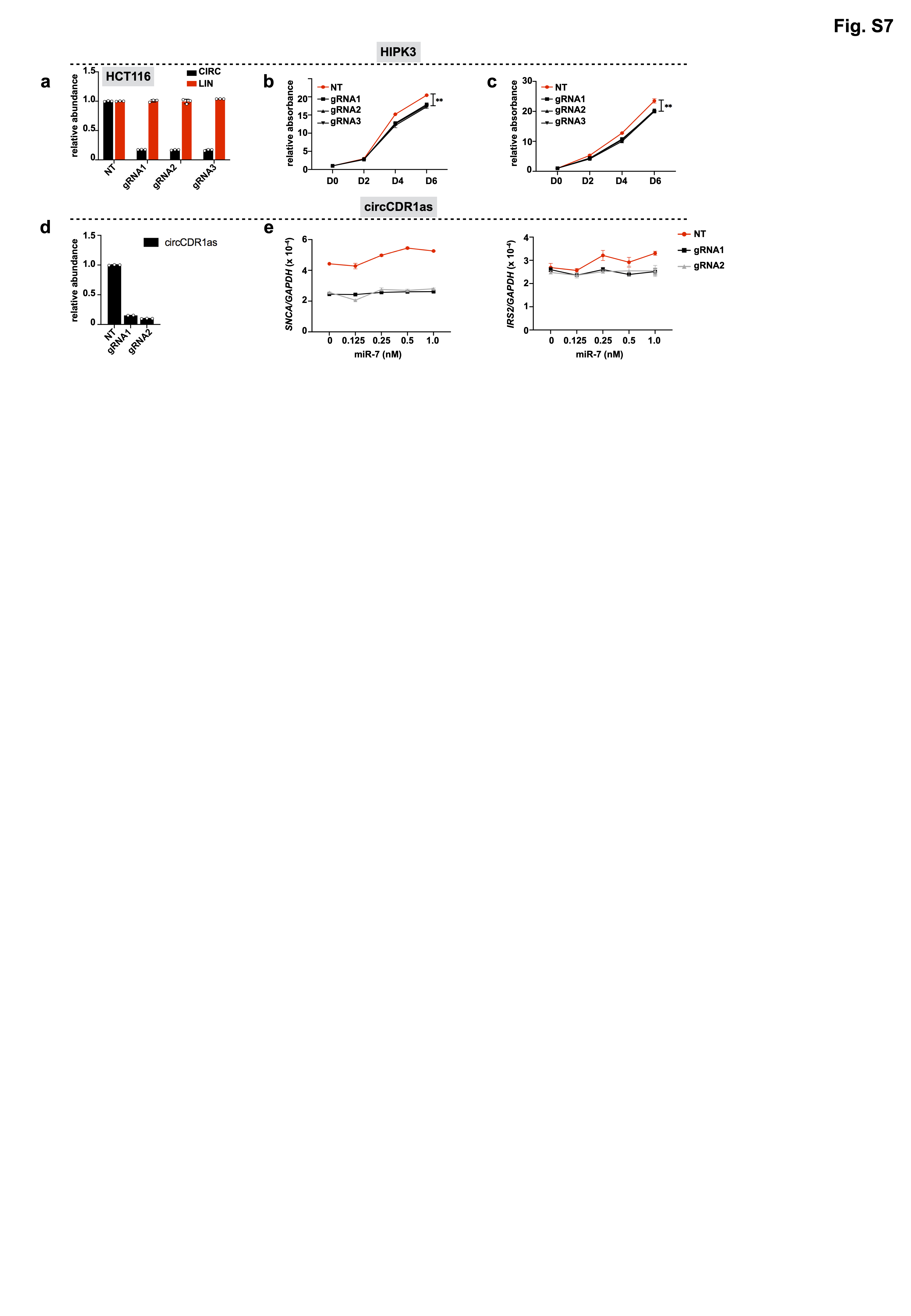
**

**Fig. S7 | Cas13d identified *bona-fide* functional circRNAs. (a)** Relative expression levels of circHIPK3 and its parental mRNA upon knock-down of circHIPK3 by gRNAs in HCT116 cells. **(b)** Proliferation rates of control and circHIPK3-silenced HCT116 cells. The number of cells was detected upon staining with crystal violet. **(c)** Proliferation rates of control and circHIPK3-silenced HCT116 cells assessed using a CCK-8 kit at indicated days. **(d)** Relative expression levels of CDR1as upon knock-down of CDR1as by gRNAs in Huh7 cells. **(e)** qRT-PCR analysis of human SNCA mRNA and IRS2 mRNA levels in CDR1as-silenced cells (black and grey line) or control cells (red line) transfected with increasing doses of miR-7 as indicated. The data shown are from one of two biological replicates with similar results, and error bars indicating the mean ± s.d. of three technical replicates. **p* < 0.05, ***p* < 0.01, ****p* < 0.001 (unpaired student’s *t* test). ns, not significant.


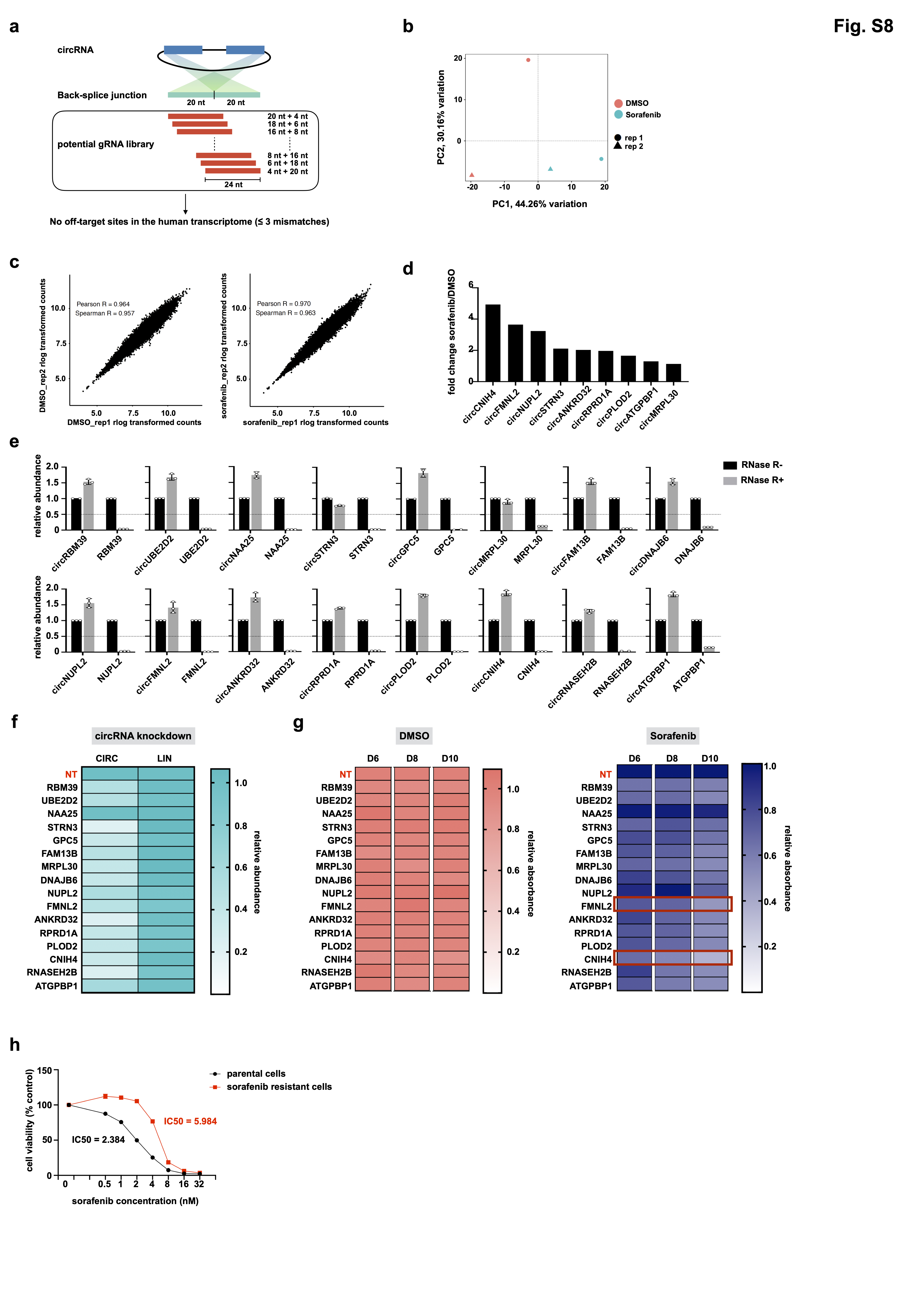


**Fig. S8 | Validation of Cas13d drug selection screening. (a)** Schematic drawing of gRNA libraries (see details in Methods). Multiple gRNAs were designed to target the BSJ sites of each of the 2,543 circRNAs. 150 non-human genomic sequence targeting gRNAs were included as negative controls. **(b)** Principal component analysis of gRNA levels across the four generated sequencing libraries for the drug selection screening. PC, principal component; rep, replicate. **(c)** The Pearson correlation coefficient between the levels of gRNAs in biological replicates of DMSO Day 14 and sorafenib Day 14 for Cas13d drug selection screening. Rep, replicate. **(d)** Fold change of circRNA candidates in sorafenib-resistant cells compared to vehicle-treated cells according to the published RNA-seq data in Wu et al. **(e)** RNase R validation of 16 selected circRNAs. circRNAs together with the corresponding linear mRNAs were amplified by qRT-PCR from cDNA prepared from RNA non-treated or treated with RNase R. **(f)** Heatmap display of the relative expression levels of circRNA candidates and their parental mRNAs upon knock-down of each circRNA. **(g)** Heatmap display of the relative cell proliferation of control and Cas13d-mediated circRNA-silenced Huh7 cells treated with either DMSO or sorafenib. Cell proliferation was measured by crystal violet staining, and absorbance at 590 nm of the treated cells from each group (Day 6, Day 8, Day 10) was normalized to NT of the same day. NT, non-targeting control. **(h)** Sorafenib-resistant Huh7 cells had lower sensitivity to sorafenib treatment compared with parental cells. Data are presented as a percentage of control cells (treated with 0 nM sorafenib). The IC50 value was calculated by nonlinear regression analysis. The data shown are from one of two biological replicates with similar results, and error bars indicating the mean ± s.d. of three technical replicates.


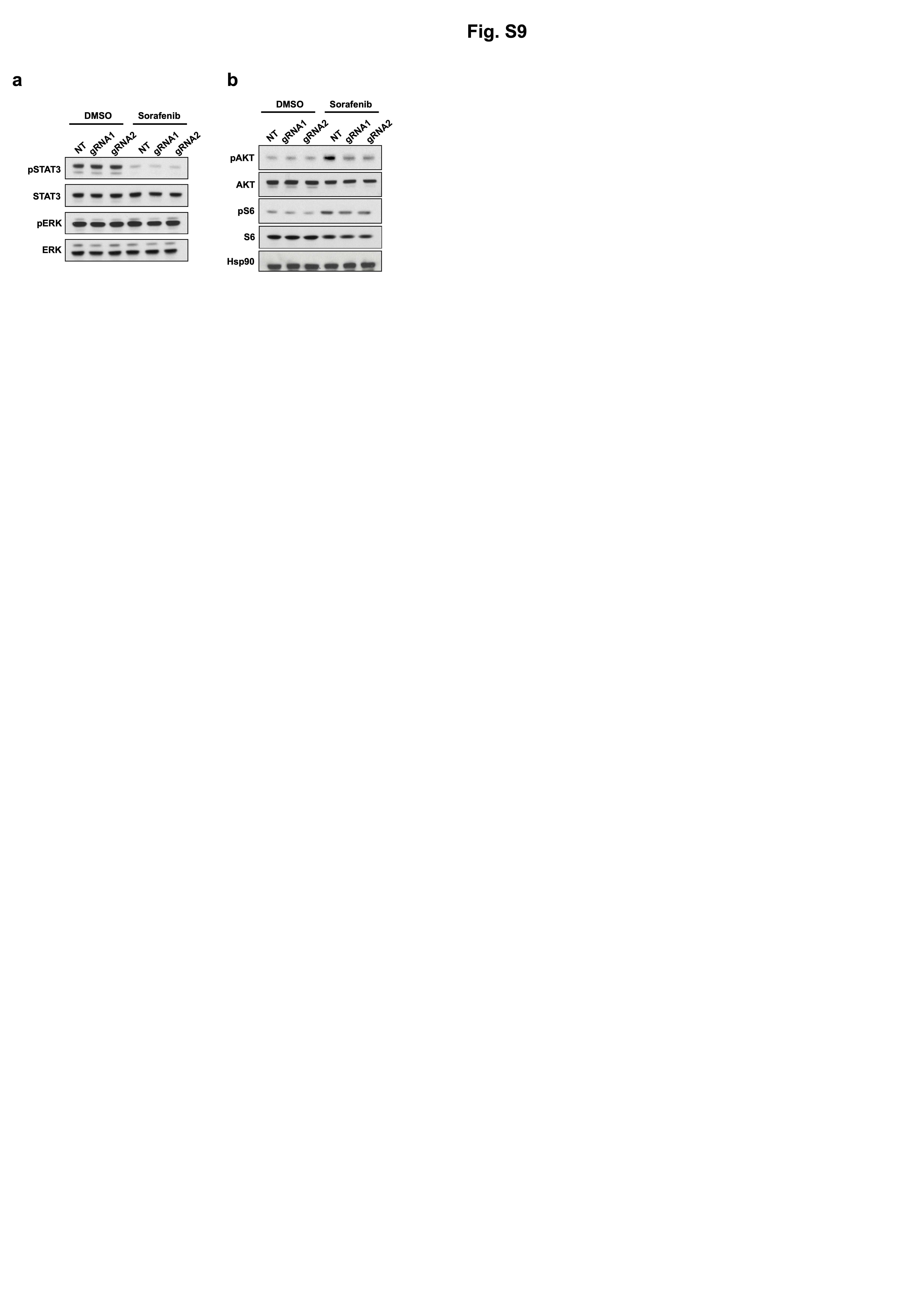


**Fig. S9 | circFMNL2 knockdown attenuates PI3K/AKT signaling pathway in sorafenib-treated cells. (a)** circFMNL2 knockdown did not affect phosphorylation of STAT3 and ERK. Total cell lysates from control and Cas13d-mediated circFMNL2-silenced Huh7 cells treated with either DMSO or sorafenib were probed with indicated antibodies. **(b)** circFMNL2 knockdown inhibited phosphorylation of AKT and S6 upon sorafenib treatment. Total cell lysates from control and Cas13d-mediated circFMNL2-silenced Huh7 cells treated with either DMSO or sorafenib were probed with indicated antibodies. Hsp90 was used as loading control.


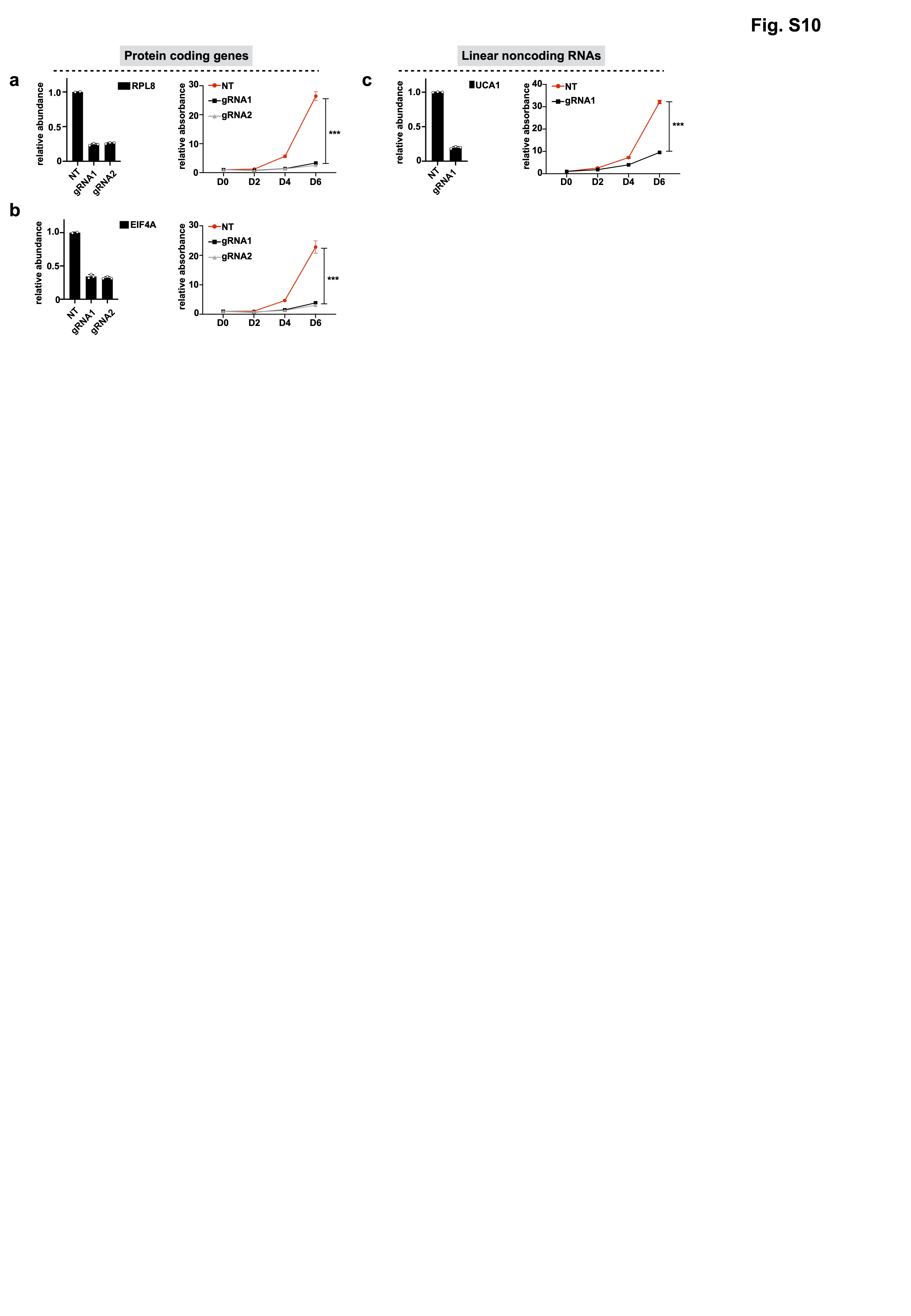


**Fig. S10 | Cas13d can also be used to investigate the function of other non-circRNA transcripts, including mRNA and lncRNA. (a)(b) Left,** relative knockdown levels of RPL8 and EIF4A by Cas13d-mediated degradation in Huh7 cells. Right, proliferation rates of control and RPL8-silenced or EIF4A-silenced Huh7 cells. The number of cells was detected by crystal violet staining. **(c)** Relative knockdown levels of UCA1 by Cas13d-mediated degradation in Huh7 cells. Right, proliferation rates of control and UCA1-silenced Huh7 cells. The number of cells was detected by crystal violet staining. The data shown are from one of two biological replicates with similar results, and error bars indicating the mean ± s.d. of three technical replicates. ****p* < 0.001 (unpaired student’s *t* test).


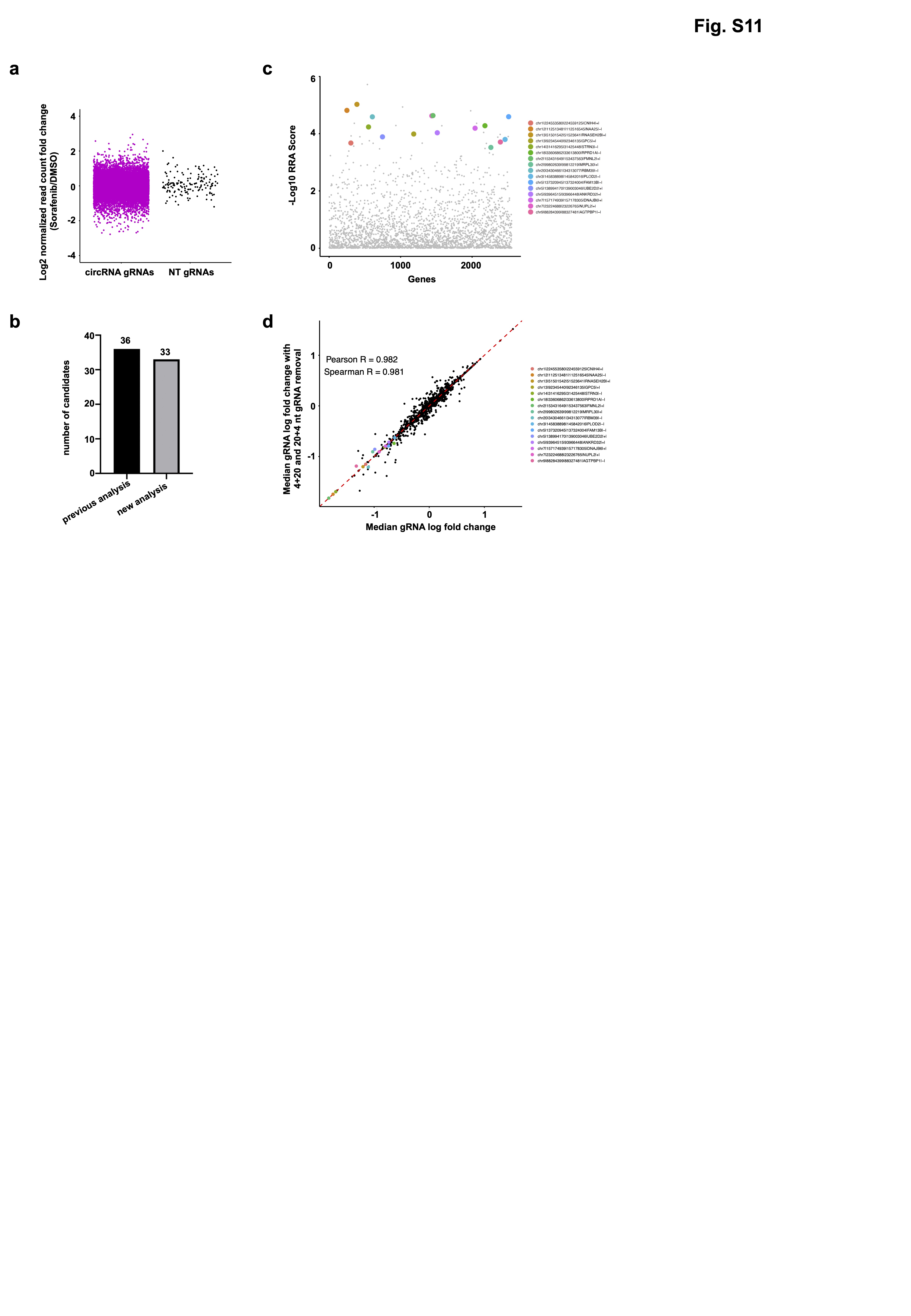


**Fig. S11 | The presence of gRNAs with the design of 4+20 or 20+4 nt does not affect screening fidelity. (a)** Plot of fold change of NT and circRNA gRNAs between DMSO- and sorafenib-treated samples. NT, non-targeting. 4+20 and 20+4 nt BSJ-gRNAs were removed from circRNA gRNAs. **(b)** Bar plot comparing the number of significant negatively selected circRNAs, as identified by MAGeCK algorithm with FDR < 0.1, without (previous analysis) and with (new analysis) the removal of 4+20 and 20+4 nt BSJ-gRNAs. **(c)** The robust rank aggregation (RRA) scores of circRNAs calculated by MAGeCK with 4+20 or 20+4 nt gRNA removal. circRNAs, which were experimentally validated in our previous analysis (without 4+20 or 20+4 nt gRNA removal) are labeled in color dots. CircRNAs are indicated with genomic locations and the host gene symbols at the end (e.g. chr12|112513481|112516545|NAA25|-|). **(d)** Scatter plot demonstrating high correlation in the median gRNA log fold change after sorafenib treatment between without (x axis) and with 4+20 or 20+4 nt gRNA removal (y axis).
