# Supplementary material for: Optimized RNA-targeting CRISPR/Cas13d technology outperforms shRNA in identifying functional circRNAs
Source: Genome Biol. 2021 Jan 21;22:41. doi: 10.1186/s13059-021-02263-9 (PMC7818937; doi:10.1186/s13059-021-02263-9)
Supplement: Supplementary file 12 — Additional file 12. Northern and Western blots. Full, uncut blots. [file 13059_2021_2263_MOESM12_ESM.docx]

**Addition file 1: Fig. S2e**, left panel, longer exposure


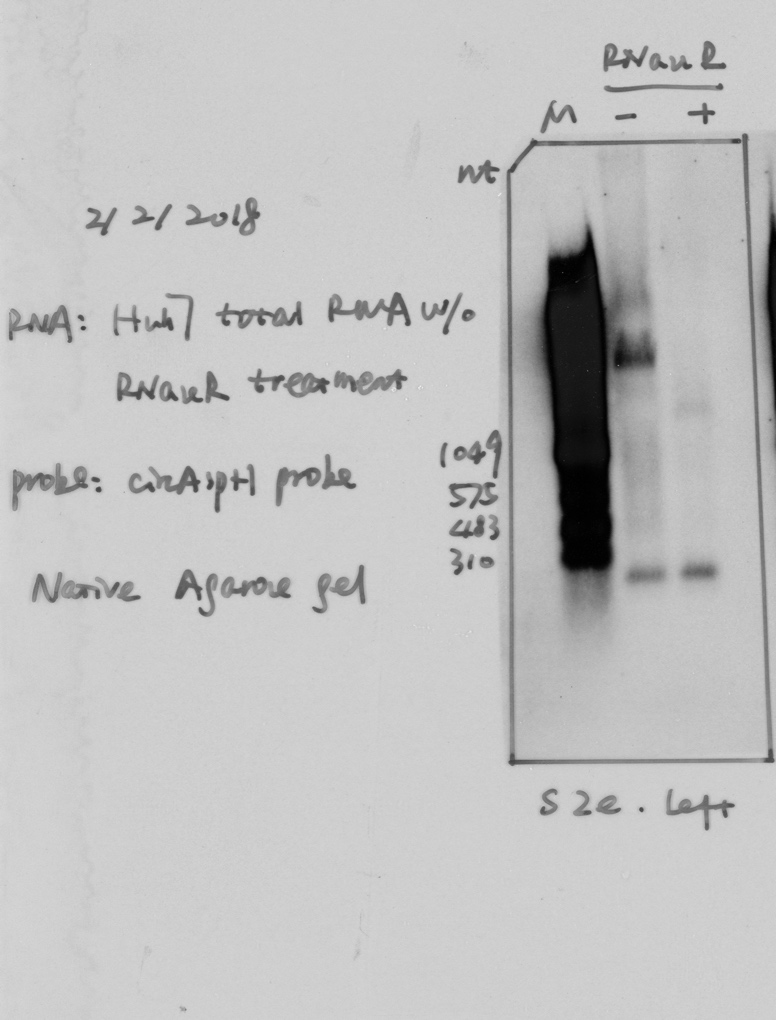


**Addition file 1: Fig. S2e**, left panel, shorter exposure for marker


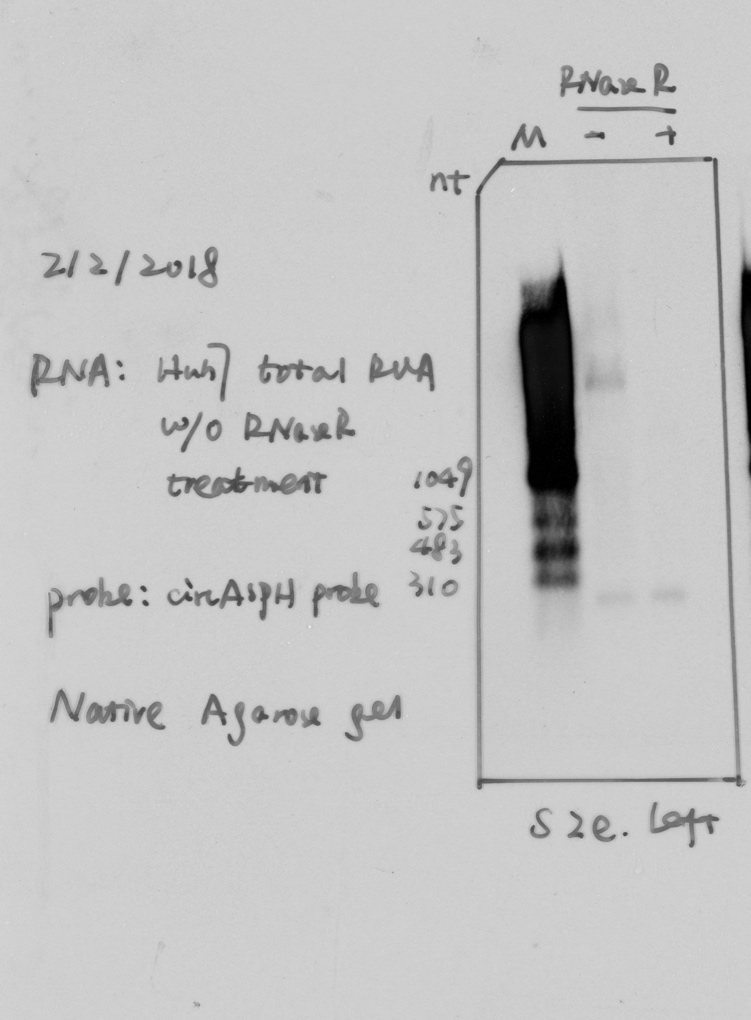


**Addition file 1: Fig. S2e**, right panel


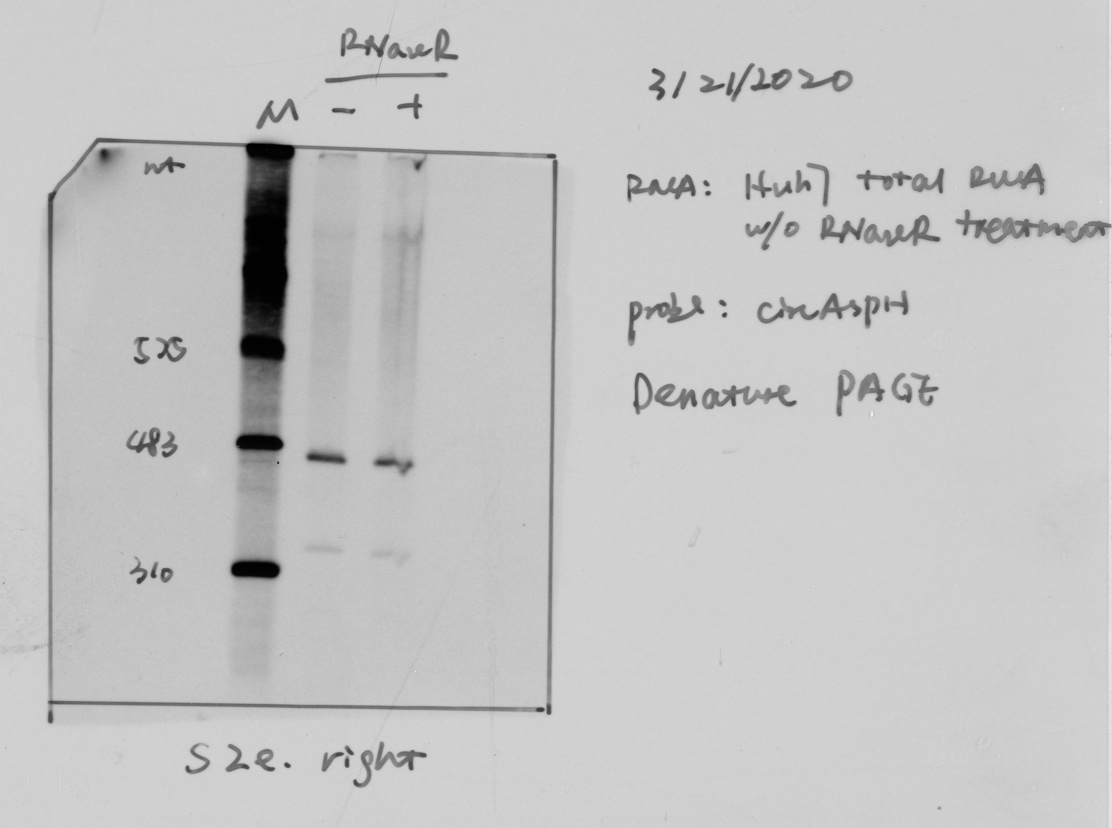


**Addition file 1: Fig. S2f**


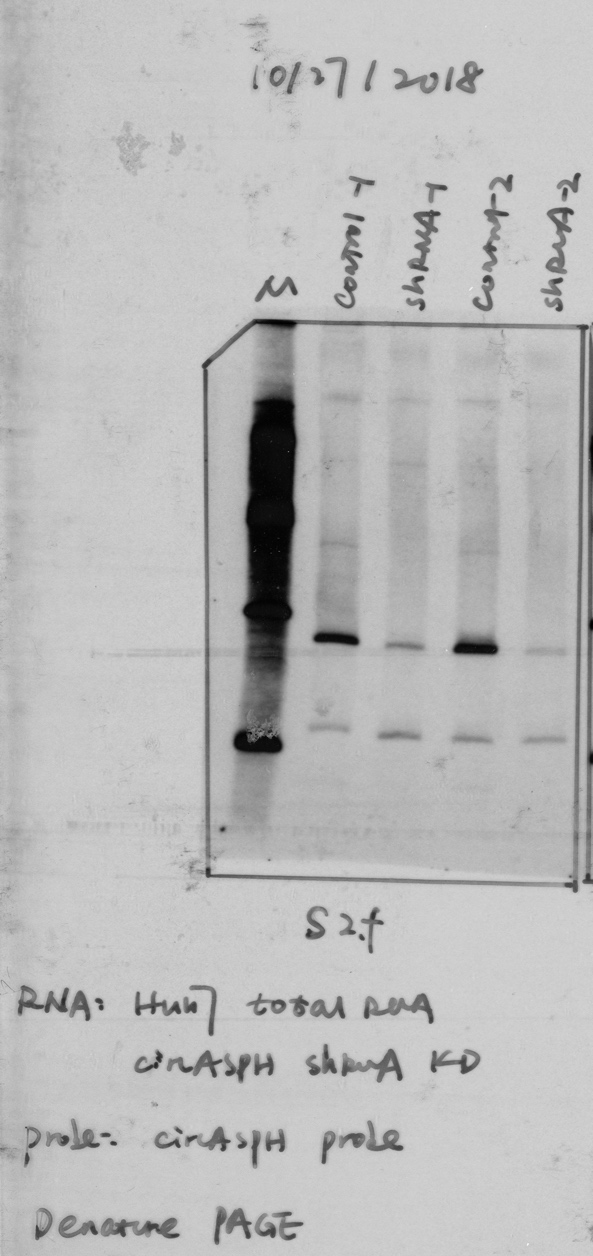


**Addition file 1: Fig. S2g**


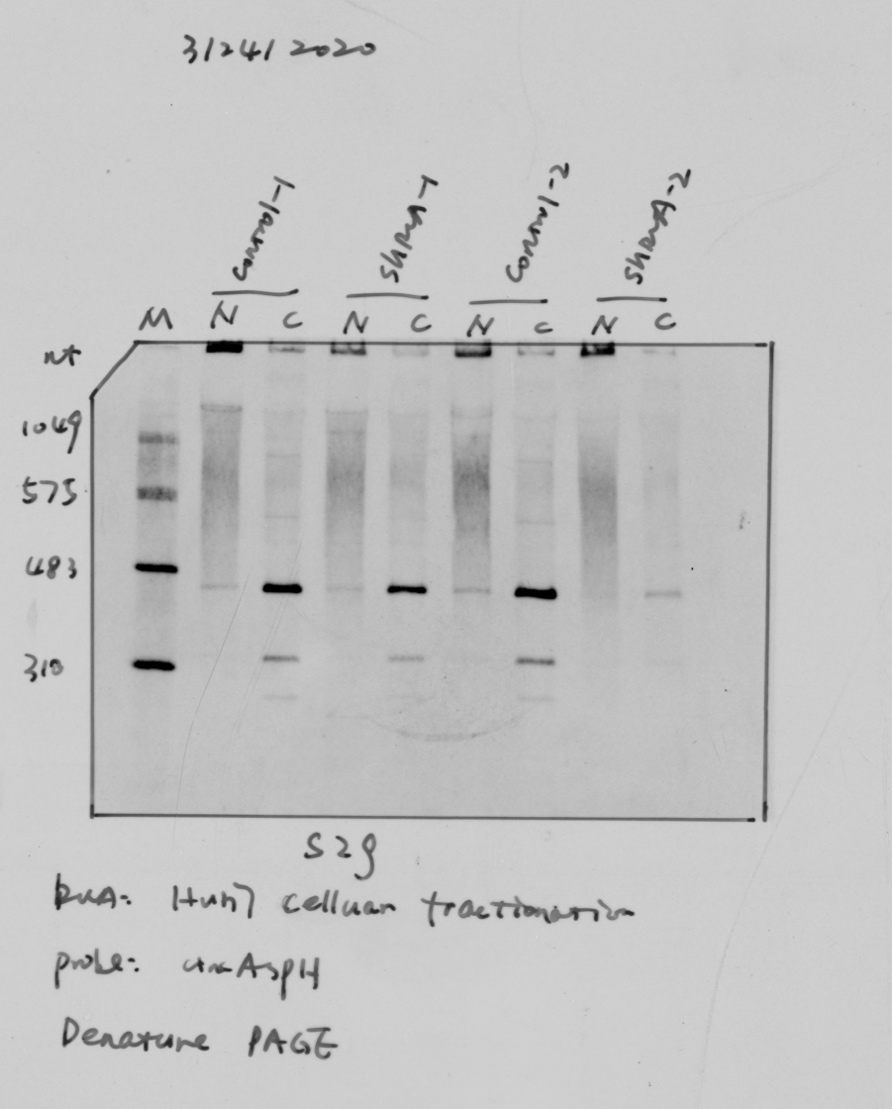


**Addition file 1: Fig. S2i**

**
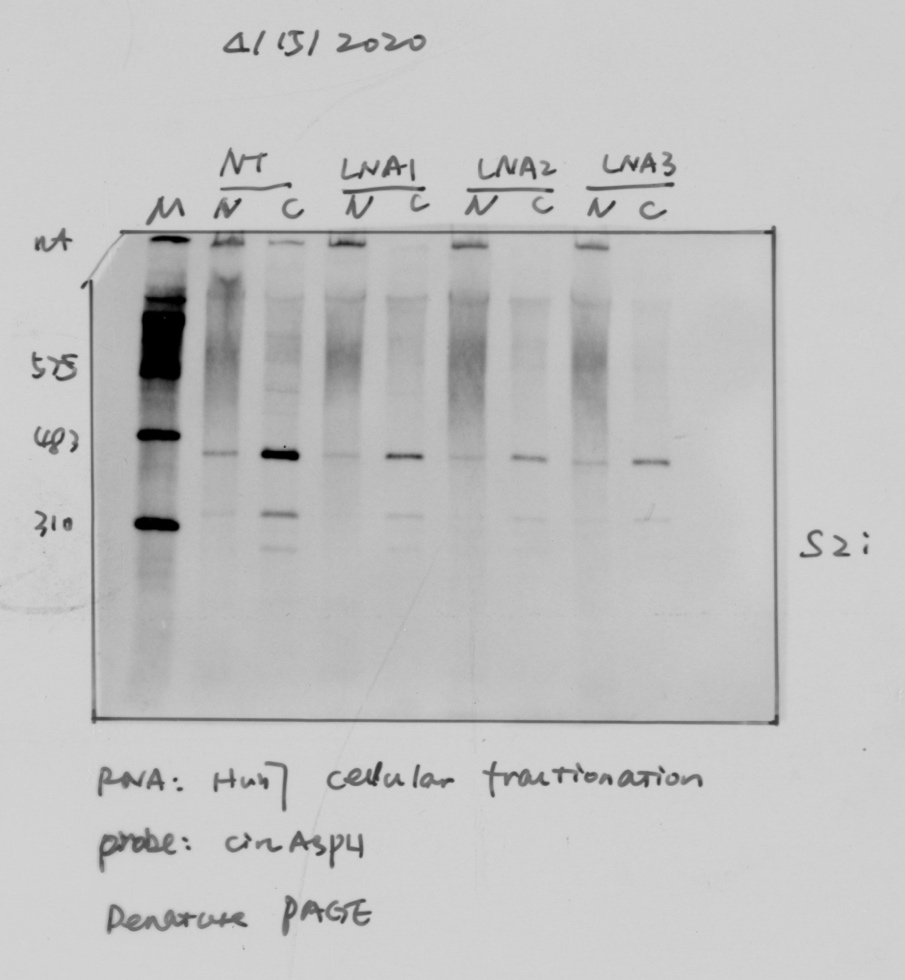
**

**Addition file 1: Fig. S2k**

**
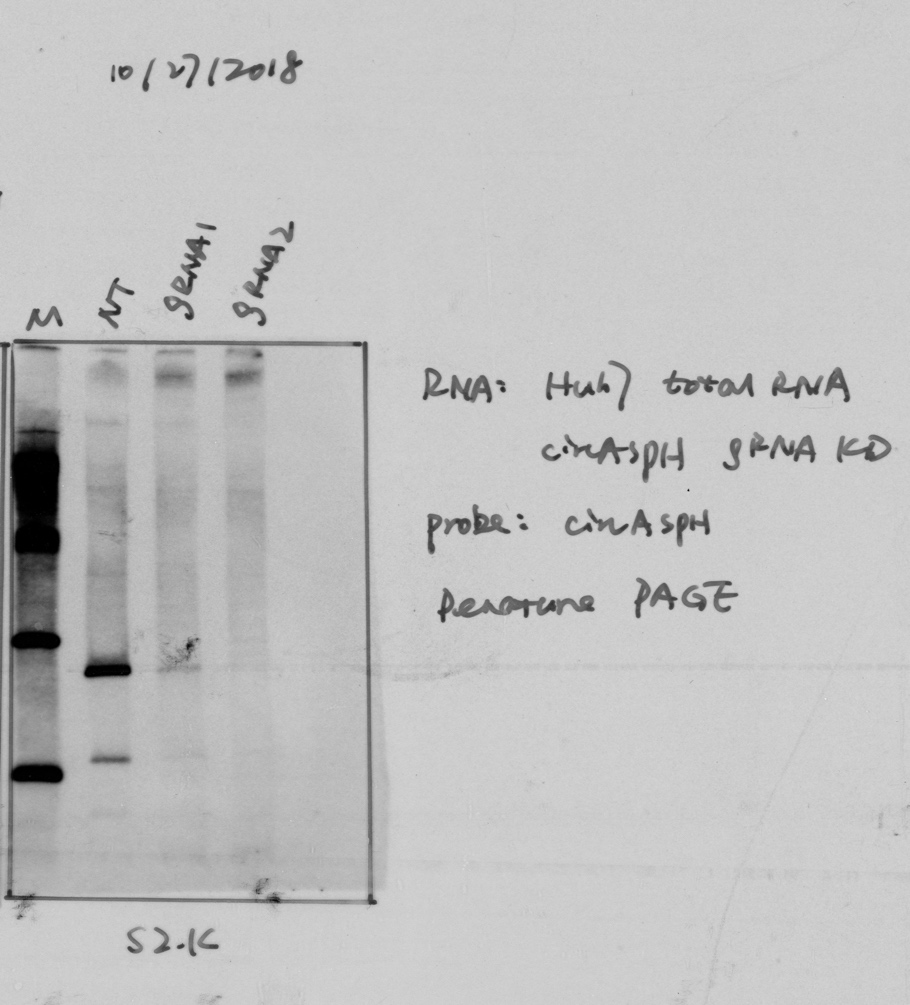
**

**Addition file 1: Fig. S2l**

**
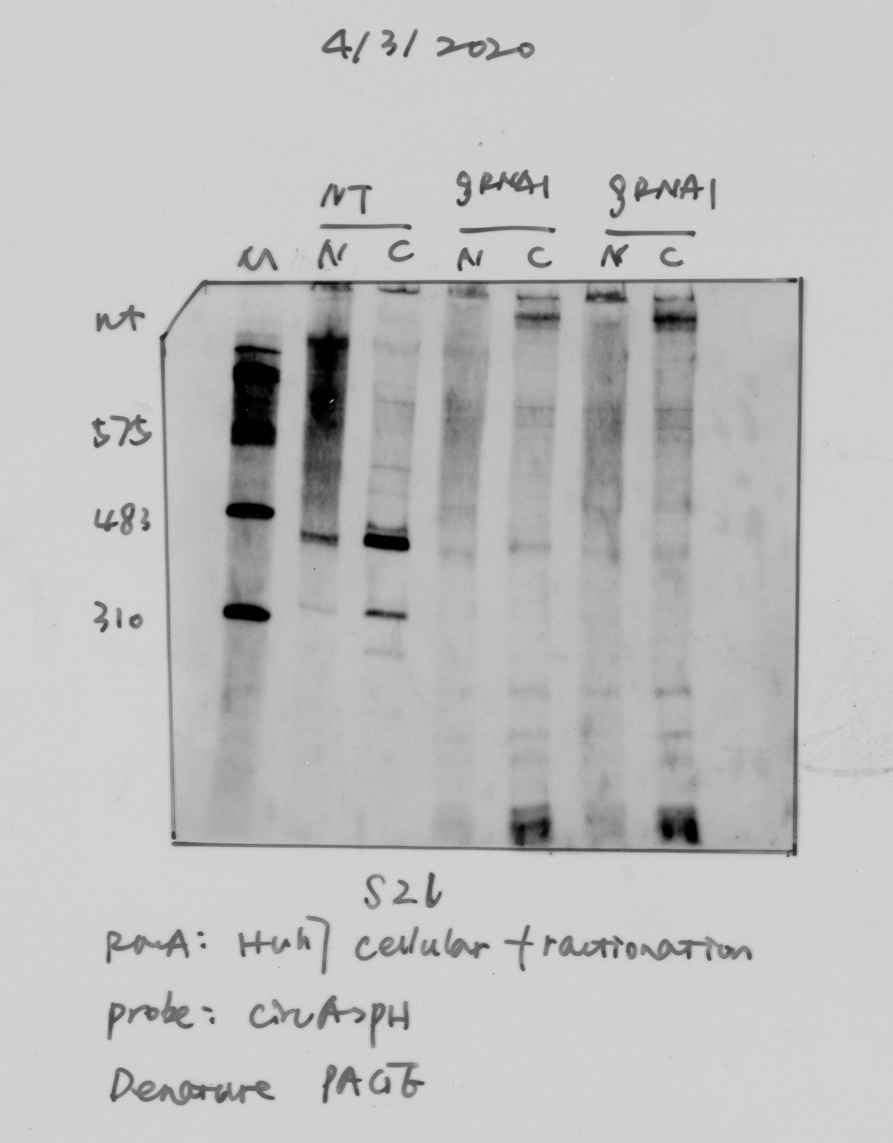
**

**Addition file 1: Fig. S3g**

**
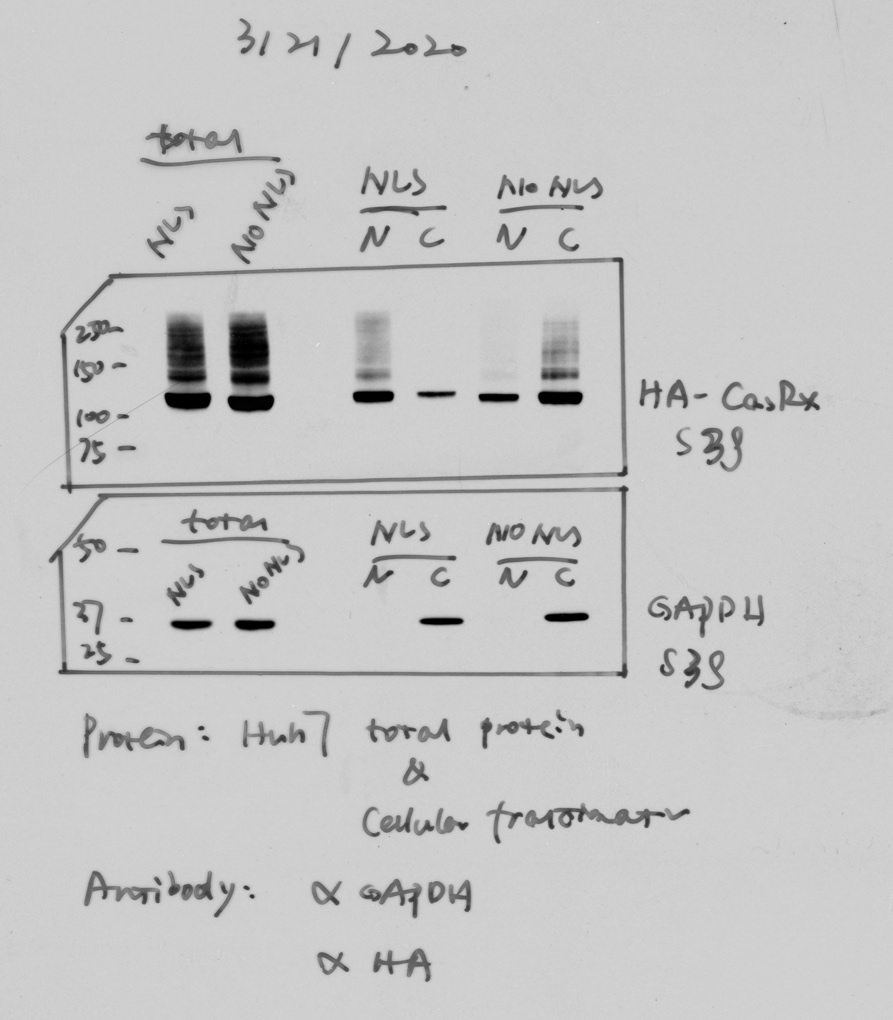
**

**
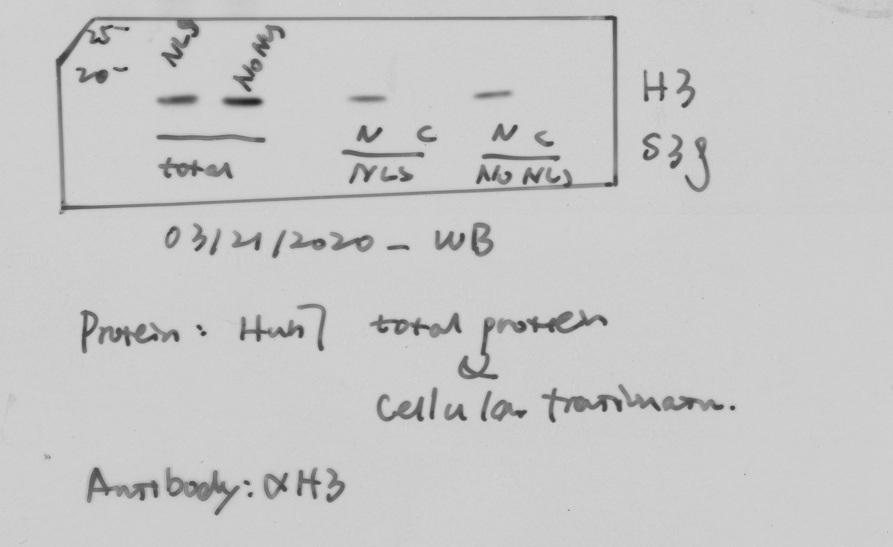
**

**Addition file 1: Fig. S9a**

**
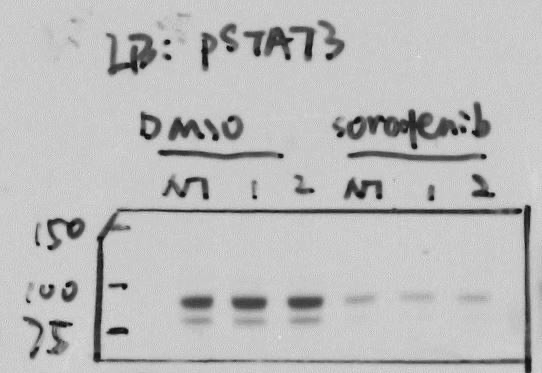

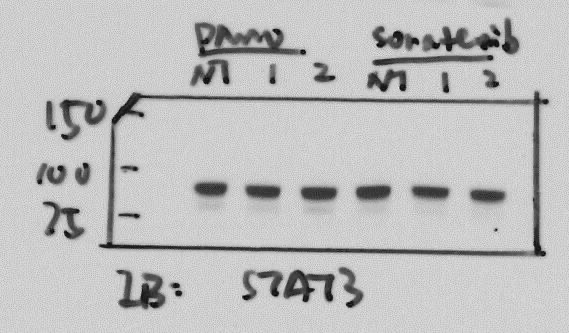
**

**
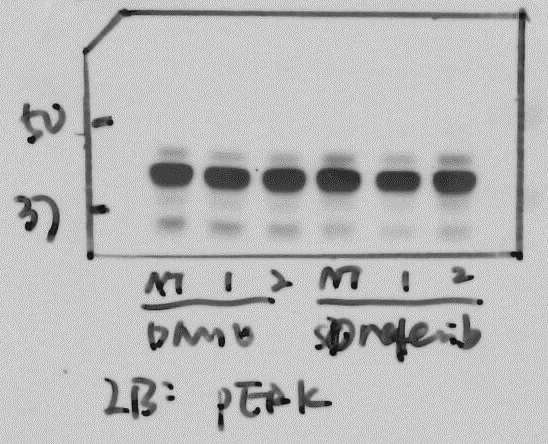

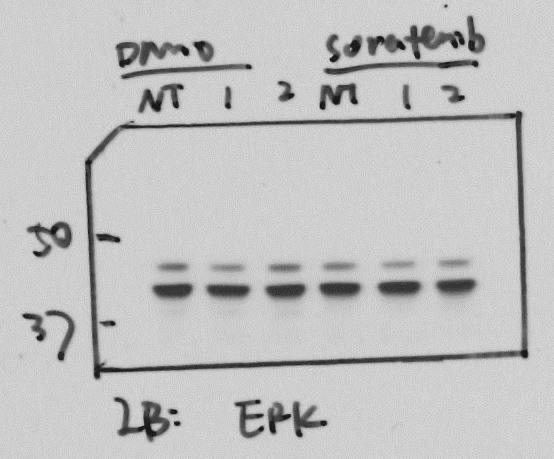
**

**Addition file 1: Fig. S9b**

**
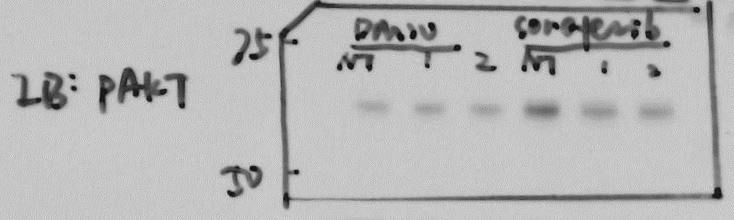

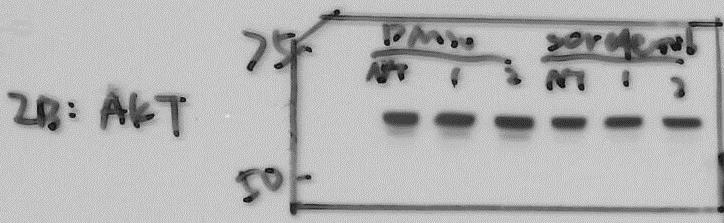
**

**
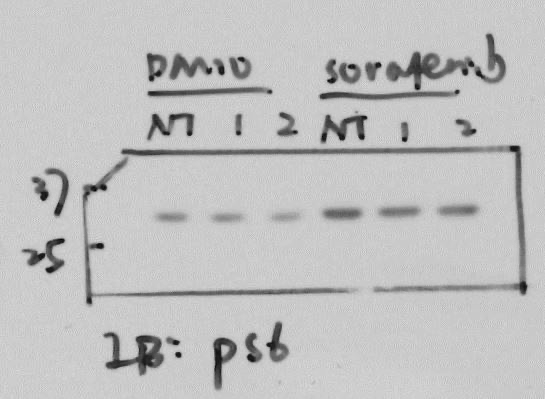

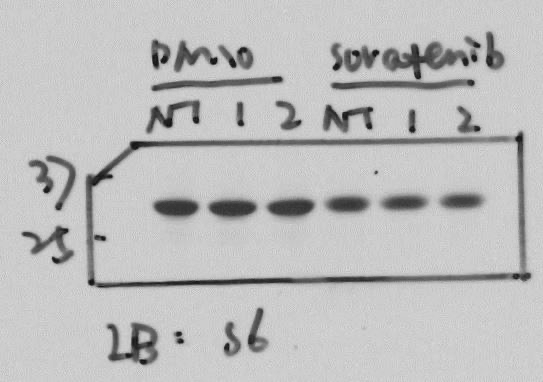

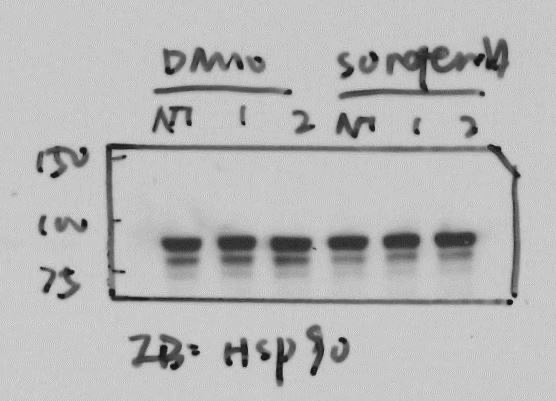
**
